# Substrate-Mimicking Peptides as MMP‑1 Inhibitors: Impact of Zinc-Binding Group Position on Ternary Complex Stability

**DOI:** 10.1021/acs.inorgchem.5c04597

**Published:** 2026-01-15

**Authors:** Paulina Potok, Wiktoria Woźniak-Laszczyńska, Robert Wieczorek, Merce Capdevila, Òscar Palacios, Elżbieta Gumienna-Kontecka, Sławomir Potocki

**Affiliations:** a Faculty of Chemistry, 49572 University of Wroclaw, 14 Joliot-Curie St., Wroclaw 50-383, Poland; b Departament de Química, Universitat Autònoma de Barcelona, Cerdanyola del Vallès (Barcelona) 08193, Spain

## Abstract

Cancer remains a leading cause of global mortality, with metastasis accounting for nearly 90% of related deaths. Matrix metalloproteinases (MMPs), and in particular MMP-1, play a pivotal role in tumor progression by degrading extracellular matrix components through a Zn­(II)-dependent catalytic mechanism. Targeting the Zn­(II) ion in the active site represents a potential approach for inhibitor design. In this study, we designed and investigated substrate-mimicking peptide inhibitors incorporating cysteine residues as zinc-binding groups (ZBGs) at distinct positions: CPQGLRG (Inh4, P4), PQGLCGR (Inh2′, P2′), and PQGLRGC (Inh4′, P4′). Using different techniques (potentiometry, mass spectrometry, NMR spectroscopy, and density functional theory calculations), we evaluated binary and ternary complexes formed between these peptides, Zn­(II), and an MMP-1 active-site model. All inhibitors formed monomeric and bis­(ligand) binary Zn­(II)-complexes, with Inh4 demonstrating the highest thermodynamic stability. In ternary systems, the MMP-1 active site model served as the primary Zn­(II) ligand coordinating through three histidine residues and reproducing the binding mode of the native enzyme. The inhibitors bound in the secondary step as the fourth coordination site, displacing the catalytic water. Ternary complexes of all inhibitors were predominant species formed above pH 6, coinciding with the optimal pH for MMP-1’s activity. Among the peptides, Inh4, which stabilized ternary complexes most effectively, coordinates Zn­(II) via its N-terminal amine. This binding mode is analogous to the strategy of tissue inhibitors of metalloproteinases. In contrast, Inh2′ and Inh4′ required structural rearrangements for Zn­(II) coordination and formed less stable complexes due to steric constraints. The findings of this study identify N-terminal cysteine as the most effective ZBG placement for stabilizing Zn­(II)-MMP-1 complexes, highlighting Inh4 as a promising lead for peptide-based MMP-1 inhibition. This work provides preliminary insights to guide the rational design of selective metalloproteinase inhibitors with therapeutic potential in cancer treatment.

## Graphical abstract

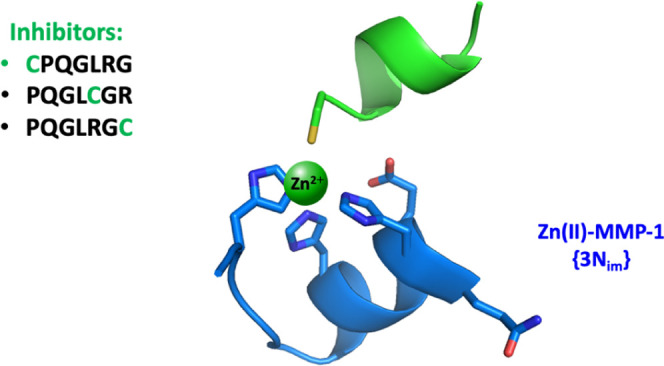

## Introduction

According to the International Agency for Research on Cancer (IARC) Organization, cancer remains a global health concern, with an estimated 9.7 million deaths reported in 2022.^1^ Moreover, by 2050, the annual new cancer cases are expected to reach 35 million, marking a 77% increase.^2^ Despite advances in targeted therapies, cancer metastasis still accounts for nearly 90% of cancer patients’ morbidity and mortality.^3,4^ Traditional treatments struggle to deliver drugs specifically to tumor cells without harming healthy tissue.^5–7^ This limitation highlights the urgent need for new therapeutic strategies that can achieve greater precision and effectiveness in targeting cancer cells.

To address these challenges, this study investigates one of the matrix metalloproteinases (MMPs) - MMP-1, a zinc-dependent enzyme whose dysregulation and overexpression are associated with cancer progression.^8,9^ MMPs degrade extracellular matrix components, by cleaving peptide bonds via the Zn­(II)-containing catalytic site, facilitating tumor invasion and angiogenesis.^10–15^ Most members of the MMP family share three conserved domains: an N-terminal propeptide, a catalytic domain, and a C-terminal hemopexin domain.^16–18^ The N-terminal propeptide contains the PRCGxPD motif, where cysteine thiol (-SH) group coordinates Zn­(II) in the catalytic domain of MMP. This cysteine-zinc bond acts as a barrier, blocking the access of catalytic water molecule from binding to the zinc ion. Proteolytic removal of this domain activates the enzyme. The catalytic domain includes a conserved Zn­(II)-binding motif HExxHxxGxxH in the active site, coordinating Zn­(II) through three histidine residues and a water molecule. The glutamate activates the coordinated water for nucleophilic attack, driving peptide bond hydrolysis (Figure 1). The C-terminal hemopexin domain further modulates substrate specificity within the MMP family.^16–20^

**Figure 1:**
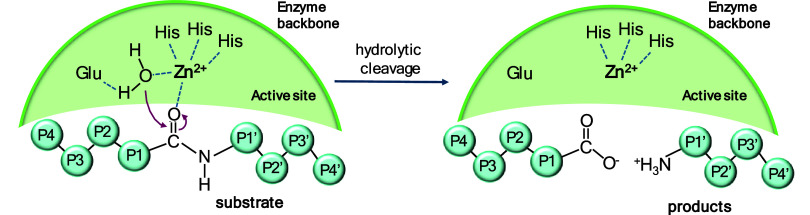
Scheme for the cleavage mechanism for MMPs.^16^

Inspired by the natural inhibition via the propeptide’s Zn­(II) coordination, we designed inhibitors to mimic this binding and maintain the blocked active site, aiming for MMP’s inhibition. Building on this concept, in the literature there are several approaches for MMP’s inhibition targeting its Zn­(II) in the active site.^14,15,21^ Synthetic MMP inhibitors (MMPIs) have been developed to chelate the zinc ion through zinc-binding groups (ZBGs), thereby displacing the nucleophilic water molecule.^21^ However, many small organic MMPIs have shown limited clinical success due to rapid clearance, poor selectivity, and low solubility.^22^ These challenges have driven interest into peptide-based inhibitors as promising alternatives with improved specificity and pharmacokinetics.^23–27^ Moreover, peptides are quite flexible in terms of chemical modifications, which is important in the field of experimental studies.^25,26^ The first designed peptide-based inhibitor was regasepin 1, a linear peptide (with the sequence PRC­(biphenylalanine)­CGE), inhibiting the MMP-8 and MMP-9, highlighting the potential of peptides as selective MMP inhibitors.^28^

Selectivity remains a critical goal in MMPI design to enhance efficacy and reduce off-target effects.^14,22^ Peptides can be engineered to mimic MMP’s substrate cleavage site sequences. These cleavage sites comprise specific amino acid motifs recognized by MMPs where peptide bonds are hydrolyzed (Figure 1).^27,29^ Table 1 summarizes representative cleavage site sequences of MMP-1, emphasizing conserved residues at positions P4 to P4’, in which between P1–P1’ MMP hydrolyzes the peptide bonds.^29–33^

**Table 1:** Representative Cleavage Sites of the Selected Matrix Metalloproteinase (Repeated Amino Acids at a Given Position among MMPs Have Been Bolded (^1^Dpa-*N*-3-(2,4-Dinitrophenyl)-l-2,3-diaminopropionic Acid)

enzyme/position	cleavage sites based on literature and MEROPS Database^12,29–33^							
	P4	P3	P2	P1	P1’	P2’	P3′	P4’
MMP-1	Lys	**Pro**	Leu	**Gly**	**Leu**	Dpa^1^	Ala	Arg
		**Pro**	**Gln**	**Gly**	Ile	Ala	**Gly**	
		**Pro**	**Gln**	**Gly**	**Leu**	Leu	**Gly**	
		**Pro**	**Gln**	**Gly**	**Leu**	Ala	**Gly**	
	Gly	**Pro**	Leu	**Gly**	**Leu**	Val	**Gly**	Gln

As part of the rational design approach used in this work, the creation of peptide-based inhibitor sequences is based on the most conserved amino acids found in these cleavage sites.^27,34^ The amino acids: proline, glycine, leucine, and glutamine are highly conserved at positions: P3 (Pro), P2 (Leu/Gln), P1 with P3′ (Gly), and P1’ (Leu), respectively (Table 1). Hence, these specific amino acids will be incorporated at these positions in the model inhibitors. For the P2 and P3′/P4’ positions, we selected glutamine and arginine to increase peptide solubility.

To enhance affinity toward the catalytic zinc­(II) ion, cysteine residue, as a ZBG has been incorporated into the peptide sequence at positions P4, P4’, or P2’ in which is not distinct preference for cleaving amino acids.^23,24^ Previous studies have shown that inhibitors containing functional ZBG in peptides targeting MMPs can improve inhibitory activity by forming a strong bond with the Zn­(II), increase selectivity to Zn­(II)-dependent MMPs and eliminate off-target interactions with other proteases.^24^

The objective of this study is to evaluate how the position of the cysteine residue as ZBG at P4, P2’, P4’ affects the formation and stability of ternary complexes with the Zn­(II)-bound active site of MMP-1. The study was conducted in two phases: first, the binary complexes formed between Zn­(II) and MMP-1’s active site, and between each peptide-based inhibitor with sequences: **C**PQGLRG (ZBG at P4), PQGL**C**GR (ZBG at P2’), PQGLRG**C** (ZBG at P4’) were investigated (Table 2); second, the ternary complexes formed by the Zn­(II)-MMP1 and these inhibitors were examined.

**Table 2:** Sequences and ZBG Positions of Ligands Analyzed in This Study

ligand	sequence	ZBG position
MMP-1’s active site	Ac-AHELGHSLGLSHS-NH_2_	N/A
Inh4	CPQGLRG	P4
Inh2’	PQGLCGR	P2’
Inh4’	PQGLRGC	P4’

Advanced techniques, including potentiometry, mass spectrometry (MS), nuclear magnetic resonance (NMR), and theoretical calculations (DFT) were employed to thoroughly analyze these complexes and determine their coordination properties and thermodynamic stability. By elucidating the influence of cysteine ZBG positioning on metal coordination and complex stability, this work provides essential insights for optimizing peptide-based MMP-1 inhibitors. These findings contribute to the development of inhibitors with enhanced selectivity and reduced off-target effects, advancing the rational design of effective therapeutic agents for cancer treatment.

## Methodology

### Materials

The tested peptides were purchased from Shanghai Royobiotech Co., Ltd., with a certified purity of 98% and were used without further modification. Their purity was assessed by potentiometric titration using the Gran method and the identity of the ligands was confirmed by mass spectrometry.^35^ Metal ion solutions were prepared using Zn­(ClO_4_)_2_ (Sigma-Aldrich) by dissolving the metal salt in filtered and doubly distilled water. The concentration of the stock solution was periodically monitored by ICP-OES. All peptide samples were prepared in a solution of 4 mM HClO_4_ (Merck). The ionic strength was adjusted to 0.1 M by adding an appropriate amount of NaClO_4_ (Merck).

### Mass Spectrometry Measurements

The ESI-TOF-MS data was obtained using a Bruker timsTOF Pro 2 instrument (Bruker Daltonics, Bremen, Germany) interfaced to a UHPLC Elute + Bruker pump and controlled using Compass software. The measurement parameters were as follows: scan range, *m*/*z* = 300–2500; dry gas 6.0 L/min; dry temperature of 90–110 °C; capillary voltage of 3500–5000 V. All spectra were processed using Bruker Data Analysis software. Mass spectra were recorded for mixtures of peptides A and L with Zn­(II): for binary systems at M:A/L molar ratios of 1:1 and 1:2, and for ternary systems at A:M:L ratios of 1:1:1 and 1:5:5. Both peptides and metal ions were dissolved in a MeOH/H_2_O solution (1:1). The pH of the peptide solution was adjusted to 7.5 before injection. The ligand concentration was 1 mM.

### Potentiometric Measurements

The protonation and Zn­(II) complex formation constants were determined from potentiometric titration curves recorded over the pH range 2–11, for samples of 2 cm^3^. The measurements were conducted using a Dosimat 665 Metrohm titrator connected to a Metrohm 691 pH meter, equipped with an InLab Semi-Micro pH electrode (Mettler Toledo). Experiments were carried out in a thermostabilized glass chamber with a magnetic stirring system, containing a microburet feed tube and an argon injection and discharge tube. Each day, the electrode was calibrated for proton concentration using HClO_4_ solution. Given the rapid kinetics of zinc ion complexation, appropriate time intervals were used between successive additions of titrant (0.1 M NaOH) to allow equilibrium to be reached. The ligand concentration in the ligand solutions was approximately 0.5 mM and the molar ratio of metal to ligand was 1:1 or 1:2. In the case of ternary complexes, the concentrations of ligands remain 0.5 mM, with the M­(II):L:A ratio 1:1:1. Experiments were conducted at a temperature of 298.2 ± 0.1 K, controlled by a circulating thermostat. To maintain inert atmosphere, high-purity argon was introduced into the solutions. Continuous magnetic stirring at a constant intensity was provided throughout the process. Calibration titrations were used to calculate the standard potential and slope of the electrode using the Glee program.^36^ The exact concentration of ligand in each sample was determined using the Gran method, while the values of the overall stability constants of the complexes were calculated using the HYPERQUAD 2008 program.^35,37^

The equilibria involved in the formation of the binary complexes may be represented as follows:

$$pM+qH+rA↔M_{p}H_{q}A_{r}$$ 1

where M is the metal ion, H is the proton, A is the ligand.

The reported log β values, pertain to the overall equilibria:

$$\log\beta =\frac{\left[M_{p}H_{q}A_{r}\right]}{{\left[M\right]}^{p}{\left[H\right]}^{q}{\left[A\right]}^{r}}$$ 2

where charges are omitted for clarity; log K_step_ values refer to the protonation process:

$$M_{p}H_{q-1}A_{r}+H↔M_{p}H_{q}A_{r}$$ 3

The stability of ternary complexes may be evaluated by the following equilibrium:

$$pM+qH+rA+sL↔M_{p}H_{q}A_{r}L_{s}$$ 4

where M is the metal ion, H is the proton, A and L are the ligands.

The global stability constant for the ternary complexes may be represented as follows:

$$\log\beta_{pqrs}=\frac{\left[M_{p}H_{q}A_{r}L_{s}\right]}{{\left[M\right]}^{p}{\left[H\right]}^{q}{\left[A\right]}^{r}{\left[L\right]}^{s}}$$ 5

where M is the metal ion, H is the proton, A and L are the ligands.

The formation of ternary complexes is defined by two possible coordination modes: simultaneous binding of both ligands to form a ternary complex:^38–40^

$$M+A+L↔\mathrm{MAL}{\log\beta }_{\mathrm{MAL}}^{M}=\frac{\left[MAL\right]}{\left[M\right]\left[A\right]\left[L\right]}$$ 6

or stepwise binding, where each ligand coordinates the metal ion at different pH values.

$$M+A↔\mathrm{MA}{\log K}_{\mathrm{MA}}^{M}=\frac{\left[MA\right]}{\left[M\right]\left[A\right]}$$ 7

$$\mathrm{MA}+L↔\mathrm{MAL}{\log K}_{\mathrm{MAL}}^{\mathrm{MA}}=\frac{\left[MAL\right]}{\left[MA\right]\left[L\right]}$$ 8

In the stepwise binding, to determine which ligand act as primary and which one as the secondary ligand *logK*_MAL_^MA^ and *logK*_MAL_^ML^ constants were calculated as follows:

$${\log K}_{\mathrm{MAL}}^{\mathrm{MA}}={\log\beta }_{\mathrm{MAL}}^{M}-\log K_{\mathrm{MA}}^{M}$$ 9

$${\log K}_{\mathrm{MAL}}^{\mathrm{ML}}={\log\beta }_{\mathrm{MAL}}^{M}-\log K_{\mathrm{ML}}^{M}$$ 10

The difference between the stability of the ternary and binary complexes shows the tendency of the formation of ternary species.^38–43^ This could be expressed by eq ([Disp-formula eq11]):

$$\Delta \log K=\log K_{\mathrm{MAL}}^{\mathrm{MA}}-\log K_{\mathrm{ML}}^{M}=\log K_{\mathrm{MLA}}^{\mathrm{ML}}-\log K_{\mathrm{MA}}^{M}$$ 11

Or eq ([Disp-formula eq12]):

$$\Delta \log K={\log\beta }_{\mathrm{MAL}}^{M}-\left(\log\beta_{\mathrm{MA}}+\log\beta_{\mathrm{ML}}\right)$$ 12

The relative stabilization (%RS) percentage allows for quantification of the stability of the ternary complex, here determined using eq ([Disp-formula eq13]):

$$\%RS=\frac{\left(\log K_{\mathrm{MAL}}^{\mathrm{MA}}-\log K_{\mathrm{ML}}^{M}\right)}{\log K_{\mathrm{ML}}^{M}}\times 100$$ 13

The constants for hydrolytic Zn­(II) species were used in calculations.^44^ The *K*a values given refer to the acid dissociation constants of the respective species. Calculated standard deviations are shown in brackets, which reflect only random errors and represent uncertainties for the last significant digit. Plots of speciation distributions were generated using the HYSS software.^45^ The pKw value was taken as 13.77 and its correctness was verified in separate experiments.

### NMR Spectroscopy

NMR experiments were performed in a magnetic field of 14.1 T induction using a Bruker Avance III 600 MHz instrument coupled to a Silicon Graphics workstation, with the sample temperature regulated to ± 0.1 K. Excitation sculpting with a 2 ms selective square pulse targeting water was used to suppress the residual water signal. Peptide samples were prepared in a mixture of 90% H_2_O and 10% D_2_O (purity 99.95%, Merck). Resonance assignments were obtained from two-dimensional ^1^H–^1^H total correlation spectroscopy (TOCSY) and nuclear Overhauser effect spectroscopy (NOESY) with standard pulse sequences. Processing and analysis of the spectra were performed using Bruker TOPSPIN 4.1, and MestreNova software. To prepare the complexes, a metal ion was added to an acidic solution containing 0.8 mM ligand at different pH values, with a total sample volume of 800 μL. In the case of inhibitors, the metal ion was titrated into the ligand (L) solution, increasing the stoichiometry from M:L = 0:1 to 1:1. For the analysis of ternary complexes, the metal ion was first added to the first ligand at an M­(II):A molar ratio of 1:1, and then the sample was gradually titrated with the second ligand to achieve a 1:1:1 ratio.

### Theoretical Calculations

Theoretical chemistry approaches, particularly molecular modeling and computational simulations, play a crucial role in elucidating the structural, electronic, and thermodynamic properties of ligands and metal complexes.^46–52^ These methods enable the prediction of stability, binding affinities, and reactivity, providing valuable insights into molecular interactions at the atomic level. In this work, we employed a hybrid ONIOM (Our Own N-layered Integrated Molecular Orbital + Molecular Mechanics) method,^53^ a multiscale quantum mechanics/molecular mechanics (QM/MM) approach, to investigate the electronic structure and molecular orbital interactions in MMP1···Zn­(II)···ligand (ligand = CPQGLRG, PQGLRGC, PQGLCGR) complexes as well as bis­(ligand) assemblies bridged by Zn­(II) ions. By combining high-level quantum chemical calculations with molecular mechanics, the ONIOM approach allows for an efficient yet accurate analysis of the electronic properties and binding behavior in these complexes, offering a deeper understanding of their stability and potential applications in catalysis, materials science, or medicinal chemistry.

The ONIOM framework divides the system into multiple layers, each treated with an appropriate level of theory, balancing accuracy and computational efficiency. Here, we used a two-layer scheme:

- The high-level region (Zn­(II) ion and its coordinating fragments) was treated with density functional theory (DFT) using the ωB97X-D functional a long-range corrected hybrid DFT method with dispersion corrections^54^–and the 6–311G­(d,p) triple-ζ basis set for improved accuracy.
- The low-level region (remaining structural components) was modeled with the AM1 semiempirical method.^55^

To prevent valency mismatches at layer boundaries, capping hydrogen atoms were introduced following standard ONIOM protocols, with C–H bond lengths fixed at 0.723886 × C–C bond length. Initial ligand geometries were derived from amino acid sequences and pre-equilibrated via 75 ps molecular dynamics (MD) simulations at 300 K using the CHARMM force field (BIO+) with a no-cutoff scheme for long-range interactions. All quantum mechanical calculations were performed in Gaussian 16 (Rev. C.01).^56^ Structures were fully optimized without constraints, and vibrational frequency analysis confirmed that all complexes reside at local energy minima, ensuring thermodynamic stability.

## Results and Discussion

### Acid–Base Properties

The peptide corresponding to the catalytic domain of **MMP-1** contains four groups able to deprotonate in the studied pH range. As this ligand represents only a fragment of the full enzyme, both the N-terminus and C-terminus are protected, and the ligand can therefore be described as H_4_L species in its fully protonated form. In contrast, the three inhibitors - **Inh4, Inh2’, and Inh4’** possess three groups able to deprotonate (H_3_L): a cysteine thiol group, an unprotected N-terminal amine, and a C-terminal carboxyl group. The deprotonation constants are provided in Table 3. The most acidic moieties are the carboxyl groups. Their p*K*_*a*_ range from 1.38 to 4.22, with the terminal −COOH being more acidic than the Glu side chain. The histidine residues of **MMP-1** ligand exhibit p*K*_*a*_ values between 6.10 and 7.56, consistent with values reported for other metalloproteinases’ catalytic domains in the literature.^57,58^ For all inhibitors, the next p*K*_*a*_ value corresponds to the N-terminal amine group, ranging from 6.73 to 8.17. The most basic moieties are the thiol group from cysteine with variations from 9.02 to 9.83 (Table 3). The variations in the deprotonation constants of the -SH, – NH_3_^+^, and – COOH groups across **Inh2**, **Inh4**, and **Inh4’** are directly influenced by the position of the cysteine residue. In **Inh4’**, where cysteine is located near the negatively charged carboxyl group, the formation of a thiolate anion is electrostatically disfavored, leading to a higher p*K*_*a*_ (9.83) and a lower p*K*_*a*_ of the carboxyl group (1.38). In contrast, when cysteine is positioned farther from the C-terminus, as in **Inh4** and **Inh2′**, the thiol group deprotonates more easily (p*K*_*a*_ **=** 9.02 and 9.21, respectively) (Table 3). A similar effect is observed for the ammonium group: when the thiol is close to the N-terminus, as in **Inh4**, the **amine** p*K*_*a*_ is lowered to 6.73. When cysteine is located farther from the N-terminus (**Inh2** and I**nh4′**), the p*K*_*a*_ values increase to **8**.17 and 8.16, respectively. The influence of cysteine on the protonation behavior of nearby functional groups is consistent with previously reported results for short peptides and dipeptide models.^59^

**Table 3:** Stability Constants (logβ) and Deprotonation Constants (p*K*_a_) Values for Analyzed Peptides and Their Zn­(II) Complexes in an Aqueous Solution of 4 mM HClO_4_ with *I* = 0.1 M NaClO_4_ at 298 K

	MMP-1 Ac-AHELGHSLGLSHS-NH_2_		Inh4 CPQGLRG				Inh2’ PQGLCGR		Inh4’ PQGLRGC			
species	logβ	p*K*_*a*_	residue	logβ	p*K*_*a*_	residue	logβ	p*K*_*a*_	residue	logβ	p*K*_*a*_	residue
HL	7.65(6)	7.65	His	9.02(2)	9.02	Cys	9.21(3)	9.21	Cys	9.83(2)	9.83	Cys
H_2_L	14.03(6)	6.38	His	15.75(3)	6.73	H_3_N^+^	17.38(3)	8.17	H_3_N^+^	17.99(3)	8.16	H_3_N^+^
H_3_L	20.13(6)	6.10	His	18.94(4)	3.19	COOH	20.14(3)	2.76	COOH	19.37(7)	1.38	COOH
H_4_L	24.35(7)	4.22	Glu									
Zn(II) complexes												
ZnHL	11.22(7)	6.10	His							14.76(5)	7.51	Cys
ZnL	5.11(2)	8.14	H_2_O	7.55(7)	8.40	H_2_O	7.13(3)	7.66	H_2_O	7.25(3)	7.85	H_2_O
ZnH_–1_L	-3.02(5)	9.37	H_2_O	-0.63(7)	10.32	H_2_O	-0.53(3)	9.70	H_2_O	-0.60(3)	9.94	H_2_O
ZnH_–2_L	-12.39(6)	9.92	H_2_O	-10.95(9)			-10.23(4)			-10.54(3)		
ZnH_–3_L	-22.31(5)											
ZnHL_2_							20.82(9)	7.91	Cys	21.52(9)	7.97	
ZnL_2_				15.58(6)			12.91(9)	9.15	H_2_O	13.55(8)	9.42	H_2_O
ZnH_–1_L_2_							3.76(10)			4.13(10)	9.63	H_2_O
ZnH_–2_L_2_				-3.69(8)						-5.50(7)	10.52	H_2_O
ZnH_–3_L_2_							-16.19(6)			-16.02(6)		

### Binary Zn­(II) Complexes

The ability of ligands to form binary complexes with Zn­(II) ions was investigated using ESI-MS. Experiments were carried out for **MMP-1** and three tested inhibitors (**Inh4, Inh2’, Inh4’**) and their Zn­(II) complexes first in a 1:1 (M:L) ratio. In the spectrum of the Zn­(II)-**MMP-1** system (Figure 2), the most intense signal (693.35 *m*/*z*, *z* = 2) corresponds to the metal-free peptide (1384.70 Da) and the next intense signal at *m*/*z* 724.31 (*z* = 2), corresponds to the [ZnL]^2+^ complex (MMP-1 + Zn^2+^ = 1446.62 Da), which indicates the formation of a 1:1 metal–ligand stoichiometry. Similar results were obtained for the Zn­(II)-**Inh4** (Figure S1), Zn­(II)-**Inh2’** (Figure S2), and Zn­(II)-**Inh4’** (Figure S3) systems, where signals at 830.32 Da confirm the formation of monomeric [ZnL]^+^ complexes. When the inhibitor is present in excess (1:2 M:L ratio), additional peaks appear corresponding to bis­(ligand) complexes. For example, in the Zn­(II)-**Inh4** system (Figure S4), signals at *m*/*z* 760.32 and 779.35 (*z* = 2), which correspond to the [Zn­(Inh4)_2_]^2+^ species and its aqua adduct respectively, confirm the formation of bis­(ligand) [Zn­(L)_2_]^2+^ complexes. The structural similarity of **Inh2’** and **Inh4’** suggests that they may exhibit comparable behavior under excess ligand conditions. The signals and isotopic distributions in the experimental and simulated spectra for selected signals are consistent, confirming the correct interpretation (Figure 2, S1–S4). Additional signals in the presented spectra are ligand/metal complex adducts and impurities left in the measuring instrument.

**Figure 2:**
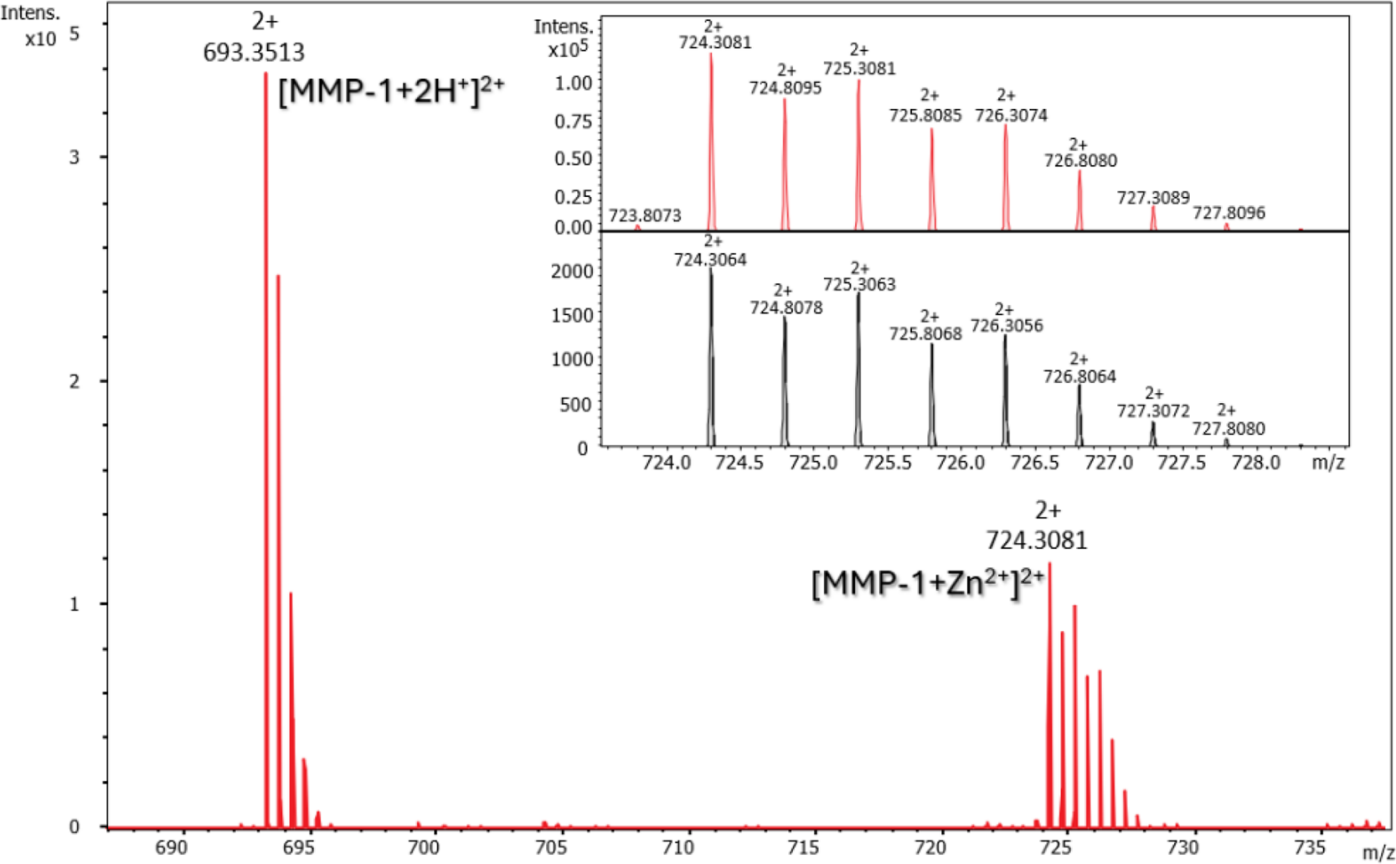
ESI-MS spectrum of a system composed of the MMP-1 ligand (L) and Zn­(II) ions in the range of *m*/*z* 685–740 (1:1 M:L). In the inset, experimental (red) and simulated (black) isotopic distribution of the signal at *m*/*z* 724.31 (*z* = 2) are presented.

The symbol L here indicates the ligand in its unprotonated form, i.e., when all the dissociable protons have been released.

### Zn­(II)-MMP-1 System

The stability constants for the Zn­(II) complexes with **MMP-1** were determined based on potentiometric titration data recorded in the pH range of 2.00–11.00 (Figure S5, Table 3). At lower pH values, the [ZnHL] species dominates, where the zinc ion is coordinated by imidazole nitrogens of two histidine residues and possibly carboxylate oxygen from a glutamic acid. As the pH increases, deprotonation of ligand leads to the formation of the [ZnL] species, which involves the coordination of a third histidine residue. This species dominates in the pH, where the full enzyme MMP-1 exhibit the maximal catalytic activity – pH 7.2–7.7.^60–62^ The coordination mode of this species was further elucidated by NMR measurements conducted at pH 7.55 (Figure S6). Upon Zn­(II) addition to the peptide solution, selective chemical shift variations on histidine residues were observed in both the aliphatic and aromatic regions. These changes confirm the direct involvement of all three imidazole side chains in Zn­(II) coordination. Additionally, chemical shift variations of signals corresponding to the glutamic acid may suggest its participation in Zn­(II) binding, its proximity to the histidine residues, or its contribution to stabilizing the complex (Figure S6). Further increases in pH lead to the formation of [ZnH_–1_L], [ZnH_–2_L] and [ZnH_–3_L] species, which arise from the progressive deprotonation of water molecules coordinated to the Zn­(II) (Figure S5, Table 3). The speciation diagram indicates that the model domain most accurately mimics the coordination environment of the native enzyme around pH 8.8, where the [ZnH_–1_L] species predominates. This species likely adopts a tetrahedral {3N_im_, H_2_O} coordination mode, with the glutamic acid either absent from the coordination sphere or playing only a stabilizing role through outer-sphere interactions.

### Zn­(II)-Inhibitor Systems

All three inhibitors can form both monomeric and bis­(ligand) Zn­(II) complexes. The first species in the monomeric complexes for **Inh4** and **Inh2’** is [ZnL], and for **Inh4’** is [ZnHL]. In the [ZnL] species, Zn­(II) is likely coordinated by the cysteine residue and the N-terminal group of all inhibitors (Figure S7A-9A, Table 3). This assignment is supported by the obtained stability constants for [ZnL] (log β = 7.13–7.55), consistent with Zn­(II) coordination involving cysteine and a second atom donor such as an amine nitrogen (Table 3).^59^ The enhanced stability constant of the [ZnL] species for **Inh4** can be attributed to the proximity of the thiol to the amine group (Table 2). In the case of **Inh4’**, due to steric preferences, coordination by a thiol and a carboxyl group cannot be excluded. The next forms observed for all inhibitors are [ZnH_–1_L], and [ZnH_–2_L] which can be assigned to the deprotonation of coordinated water molecules (Figure S7A -9A, Table 3).

All inhibitors also form bis­(ligand) Zn­(II) complexes, with the first species [ZnHL_2_] for **Inh2’** and **Inh4’**, and [ZnL_2_] for **Inh4** (Figure S7B-9B). The [ZnL_2_] species are characterized by logβ values of 15.58 for **Inh4**, 12.91 for **Inh2′**, and 13.55 for **Inh4′** (Table 3). These values can be interpreted by comparison with model peptides containing a single cysteine. **Inh4** likely coordinates Zn­(II) through 2S^–^ and 2NH_2_ groups, based on the similarity of its stability constant to that of the Ac-HAAC-NH_2_ ligand (log β = 13.58 for binding mode 2S^–^, 2N_im_). **Inh2′**, which exhibits the lowest stability, most likely binds Zn­(II) via 2S^–^ donors only, comparable to Ac-AAAC-NH_2_ (log β = 9.72). The coordination mode of **Inh4′** probably involves 2S^–^ and 2COO^–^ donors, as seen in Ac-CGAD-NH_2_ (log β = 10.3), or as for **Inh2’** only through two thiol groups.^63,64^ The deprotonation of water molecules leads to the formation of the [ZnH_–2_L_2_] for **Inh4** and **Inh4’**, and [ZnH_–3_L_2_] for **Inh2’** and **Inh4’** ligands (Figure S7B-9B).

To evaluate the tendency for monomeric and bis­(ligand) complex formation, log­(K_1_/K_2_) values were calculated (Tab. S1).^59,65^ The negative log­(K_1_/K_2_) value for **Inh4** indicates a strong preference for bis­(ligand) complex formation. In contrast, the positive value for **Inh2’** suggests a stronger preference for monomeric complexes. **Inh4′** displays an intermediate value, indicating a moderate tendency toward monomeric complex formation.

To gain deeper insight into the coordination behavior of Zn­(II) with the inhibitors, 2D ^1^H–^1^H NMR spectroscopy was performed at pH 7.55. Spectra were recorded for each metal-free ligand and upon successive additions of Zn­(II) (0.25, 0.5, 0.7, and 1.0 equiv) (Figure 3, Figure S10-11). The most notable spectral perturbations were observed in the Hα-Hβ cross-peaks of the cysteine residues, providing direct evidence of their involvement in metal coordination.

**Figure 3:**
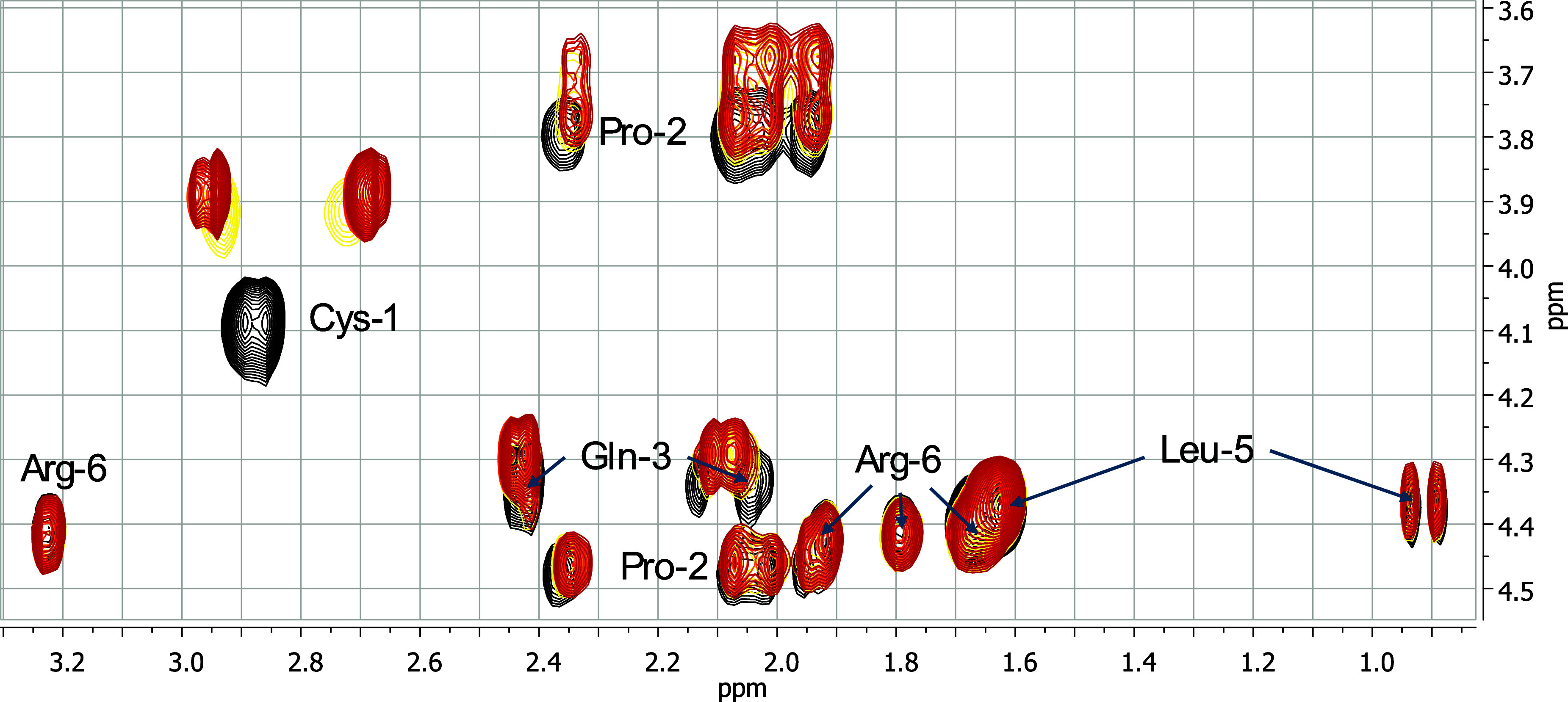
Superimposition of aliphatic regions of ^1^H–^1^H TOCSY spectra of 0.8 mM Inh4 peptide (CPQGLRG), pH 7.55, in the absence (black) and in the presence of increasing equivalents of Zn­(II): 0.25 (yellow), 0.5 (orange), 0.7 (dark orange) and 1.0 (red).

Additionally, for **Inh2’**, selective chemical shift variations for Pro-1, Gln-2, and Leu-4 protons were observed upon the addition of 0.7 and 1.0 equiv of Zn­(II) to the peptide solution (Figure S10) These perturbations suggest conformational reorganization of the peptide to accommodate Zn­(II) coordination and optimize the binding geometry. Similar effects were observed for **Inh4’** (Figure S11). In contrast, **Inh4** with cysteine at the N-terminal site, showed no such perturbations, consistent with its ability to achieve effective Zn­(II) binding via proximal thiol and amino groups without requiring structural changes (Figure 3).

The bis­(ligand) complexes were further investigated through DFT calculations. All tested inhibitors form stable bis­(ligand) complexes bridged by Zn­(II) cations, as demonstrated in Figure 4. This coordination structures creates a metallic center with metal:ligand ratios of 1:2 (Zn­(II):ligand, where each Zn­(II) ion acts as a structural node connecting two peptide units.

**Figure 4:**
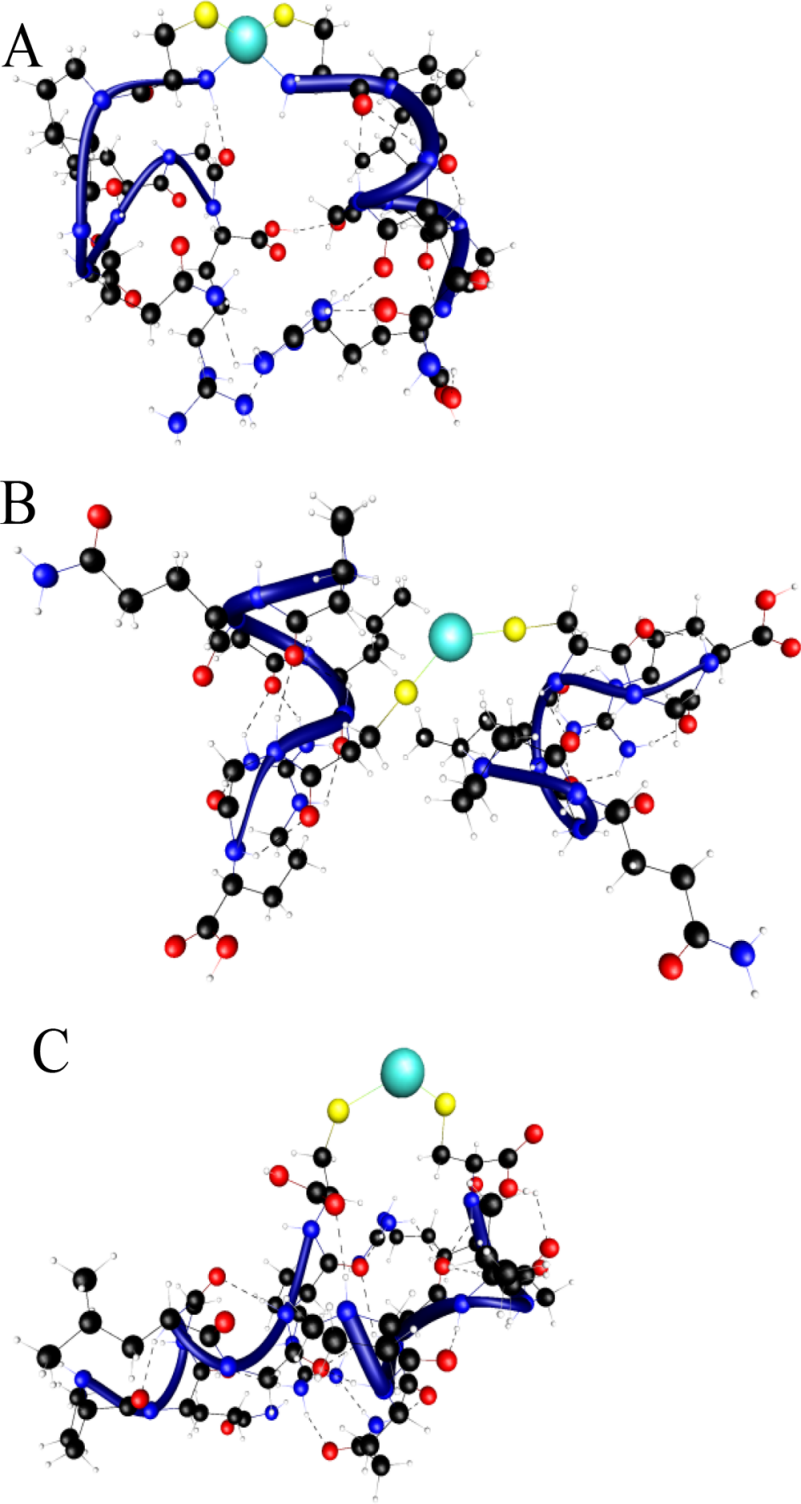
Structures of the (A) Zn­(II)···(Inh4)­2, (B) Zn­(II)···(Inh2’)­2, and (C) Zn­(II)···(Inh4’)­2 complexes.

All bis­(ligand) complexes feature a characteristic S­(Cys_A_)···Zn­(II)···S­(Cys_B_) bridging motif (Figure 4). However, the Zn­(II)···(Inh4)_2_ complex exhibits additional stabilizing interactions originating from the unique spatial arrangement of its N-terminal cysteine residues. This enhanced coordination network - combining both thiolate bridges and auxiliary N-terminal contacts - explains the superior thermodynamic stability of this particular dimer compared to others in the series Zn­(II)···(**Inh4**)_2_≫Zn­(II)···(**Inh4’**)_2_ > Zn­(II)···(**Inh2’**)_2_. Table S2 summarizes the key metal–ligand coordination distances, including Zn­(II)-S­(thiolate) and Zn­(II)-N­(amine) bond lengths, for all characterized complexes. These metrical parameters provide quantitative insight into the varying coordination patterns and their correlation with complex stability.

### Ternary Zn­(II) Complexes

ESI-MS experiments were also performed for ternary systems: **MMP-1**-Zn­(II)-**Inh2’, MMP-1**-Zn­(II)-**Inh4** and **MMP-1**-Zn­(II)-**Inh4’**. In the obtained spectra for all tested systems, an intense signal can be observed at *m*/*z* 1089.99 (z = 2), corresponding to the [MMP-1-Zn-Inh]^2+^ complexes, which indicates the formation of ternary complexes (Figure 5A). The signals and isotopic distributions in the experimental and simulated spectra for selected signal are consistent, confirming the formation of the expected complexes (Figure 5B).

**Figure 5:**
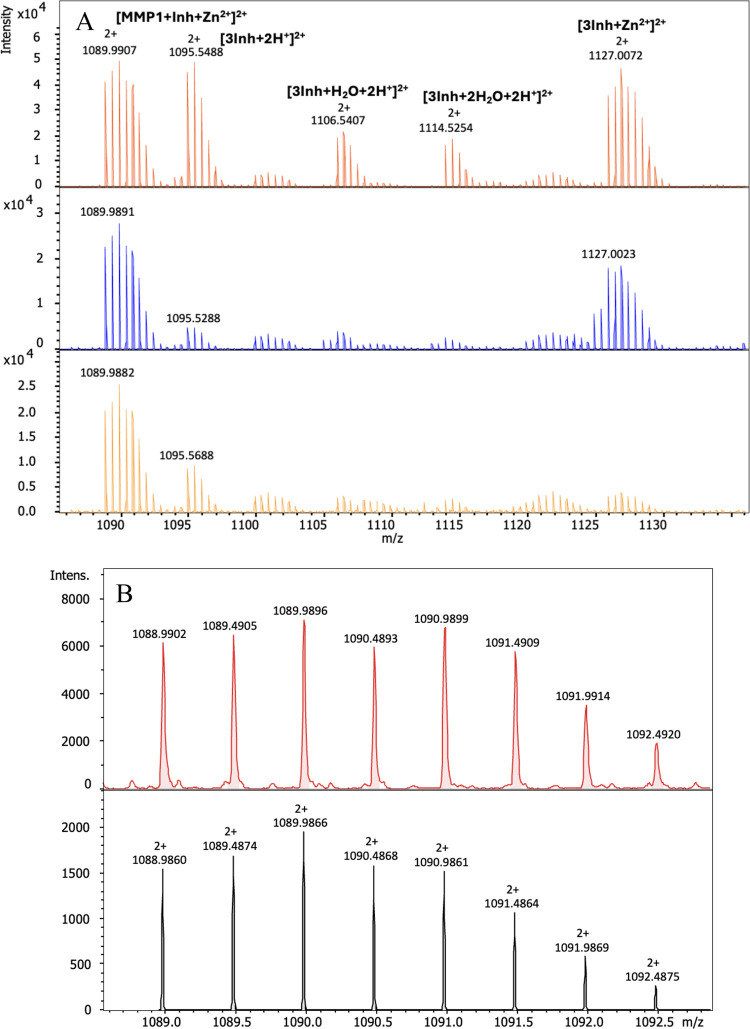
(A) ESI-MS spectra of a ternary systems in the range of *m*/*z* 1085–1140 (1:5:5 A:M:L): MMP-1-Zn­(II)-Inh2’ (orange), MMP-1-Zn­(II)-Inh4 (blue), and MMP-1-Zn­(II)-Inh4’ (yellow); (B) experimental (red) and simulated (black) isotopic distribution of the signal at *m*/*z* 1089.98 (*z* = 2), confirming the assignment of the [ZnAL]^2+^ complex.

Furthermore, direct comparison of the binary and ternary systems in Figure 6 confirms that signal at *m*/*z* 1089 (*z* = 2) corresponds to the formation of the ternary complex. The binary systems, Zn­(II)-**MMP-1** and Zn­(II)-**Inh4,** do not show any detectable ion near this range, indicating that this ion is not formed by Zn­(II) interacting with individual components alone but arises exclusively from the simultaneous interaction of **MMP-**1, Zn­(II), and **Inh4** (Figure 6). The same results as that observed for **Inh4** were obtained when analyzing **Inh2′** and **Inh4′.**

**Figure 6:**
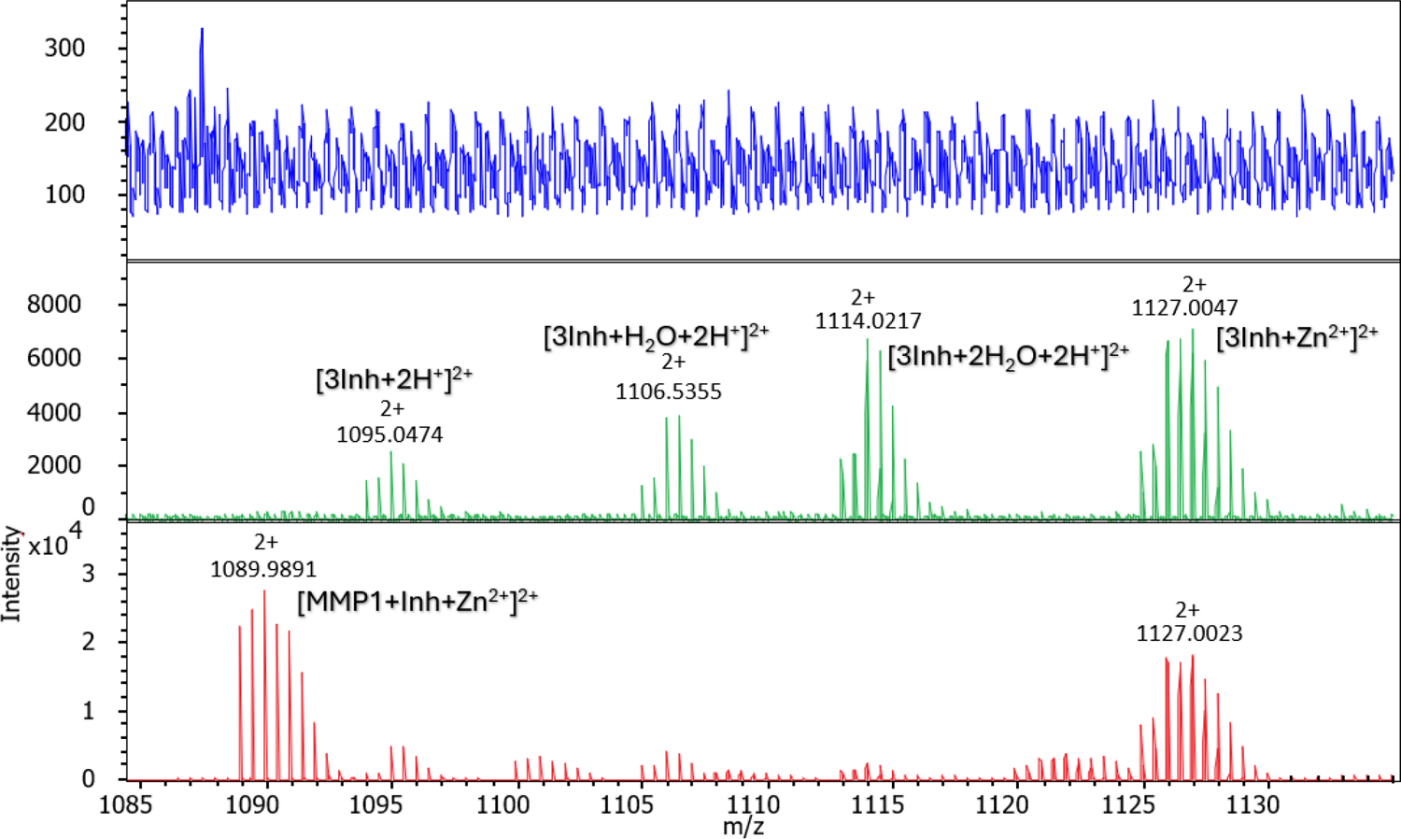
ESI-MS spectra of a binary and ternary systems in the range of *m*/*z* 1085–1140 (1:5:5 A:M:L): Zn-MMP-1­(II) (blue), Zn­(II)-Inh4 (green), and MMP-1-Zn­(II)-Inh4 (red).

Potentiometric measurements enabled the determination of ternary complex species, their stability constants (logβ), and deprotonation constants (p*Ka*) (Table 4), as well as the construction of species distribution diagrams (Figure 7, Figures S12, S13).

**Table 4:** Stability Constants for Zn­(II) Ternary Complexes Involving MMP-1 (A) and Different Inhibitors (L) in Aqueous Media at 25°C and I 0.1 mol L^–1^ NaClO_4_

	Inh4		Inh2’		Inh4’	
species	logβ	p*K*_*a*_	logβ	p*K*_*a*_	logβ	p*K*_*a*_
ZnH_2_AL	26.05(8)		25.97(6)	6.86	26.75(7)	6.76
ZnHAL			19.11(6)	7.04	19.99(5)	7.66
ZnAL	13.91(5)	8.77	12.07(3)	8.97	12.33(4)	9.03
ZnH_–1_AL	5.14(7)		3.10(5)	9.96	3.30(3)	
ZnH_–2_AL			–6.86(7)			

**Figure 7:**
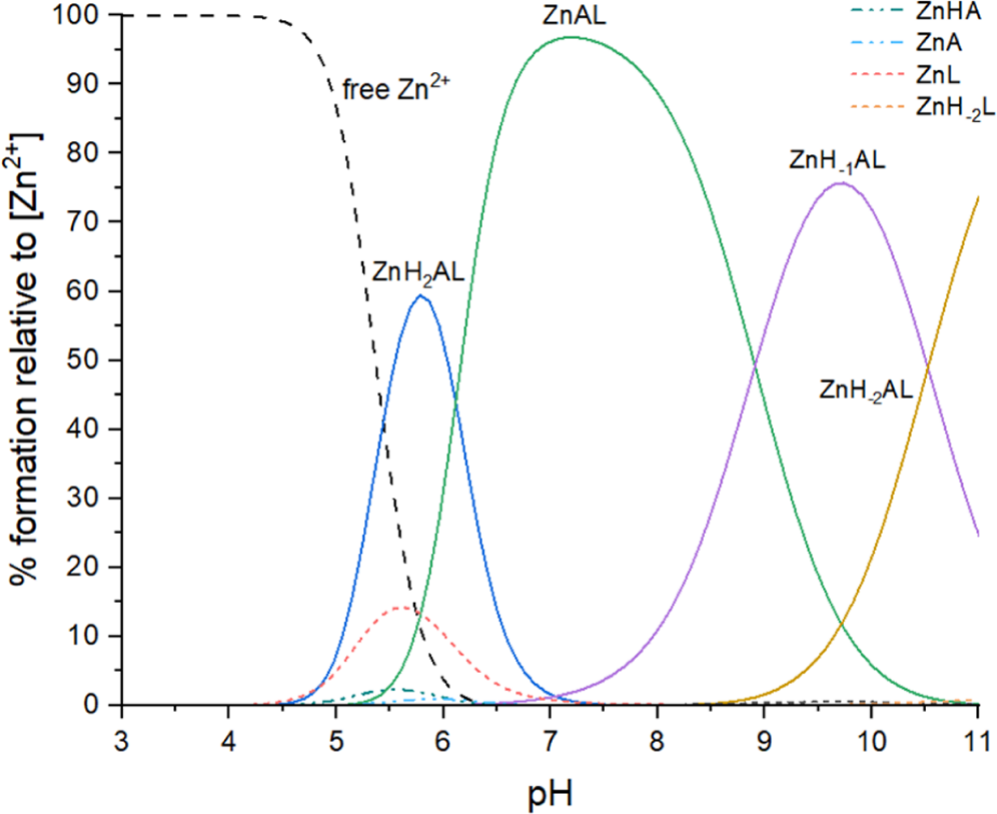
Species distribution diagram for MMP-1-Zn­(II)-Inh4 complexes (A – MMP-1, L – Inh4) in an aqueous solution; *C*_*L*_ = 0.5 mM; Zn­(II):A:L = 1:1:1.

### MMP-1-Zn­(II)-Inh4 System

Ternary complex formation begins at pH 4.5, with the ZnH_2_AL species (A = **MMP-1**, L = **Inh4**). At this pH, binary species ([ZnHA] and [ZnL]) are also present (Figure 7). The next ternary species, [ZnAL], reaches its maximum concentration at around pH 7.7. As the pH increases, successive deprotonation of coordinated water molecules leads to the formation of [ZnH_–1_AL] and [ZnH_–2_AL] species, further stabilizing the complex (Table 4, Figure 7). As shown in the distribution diagram, from pH 6 onward, only ternary species are present, with no evidence of binary complexes. This range overlaps with the pH, where **MMP-1** displays maximal catalytic activity (pH = 7.2–7.7).^60–62^ It suggests that **Inh4** may be a promising inhibitor of **MMP-1** enzymatic activity by binding to the Zn­(II)-**MMP-1** complex and altering the metal coordination sphere. The structural characterization of the **MMP-1**-Zn­(II)-**Inh4** ternary system was further investigated by 2D ^1^H–^1^H TOCSY NMR spectroscopy at pH 7.55, where ZnAL species predominate (Figure 8). The spectra show new resonances that do not overlap with those of the binary Zn­(II)-**MMP-1** or Zn­(II)-**Inh4** complexes, confirming the formation of a new coordination environment. Histidine residues from **MMP-1** exhibit a variation in their Hα-Hβ correlations compared to binary Zn­(II)-**MMP-1** complex (Figure 8), supporting their direct involvement in Zn­(II) binding within a modified coordination environment. Moreover, Glu-1 side-chain protons shift toward the free-ligand positions, suggesting a reduced contribution of the carboxylate group in the ternary system. The Cys-1 Hα-Hβ cross-peaks of **Inh4** display only minor perturbations relative to the Zn­(II)-**Inh4** binary complex (Figure 8), indicating that the local Zn­(II) environment around cysteine remains largely similar, with coordination in the ternary complex *via* the N-terminal group or thiol.

**Figure 8:**
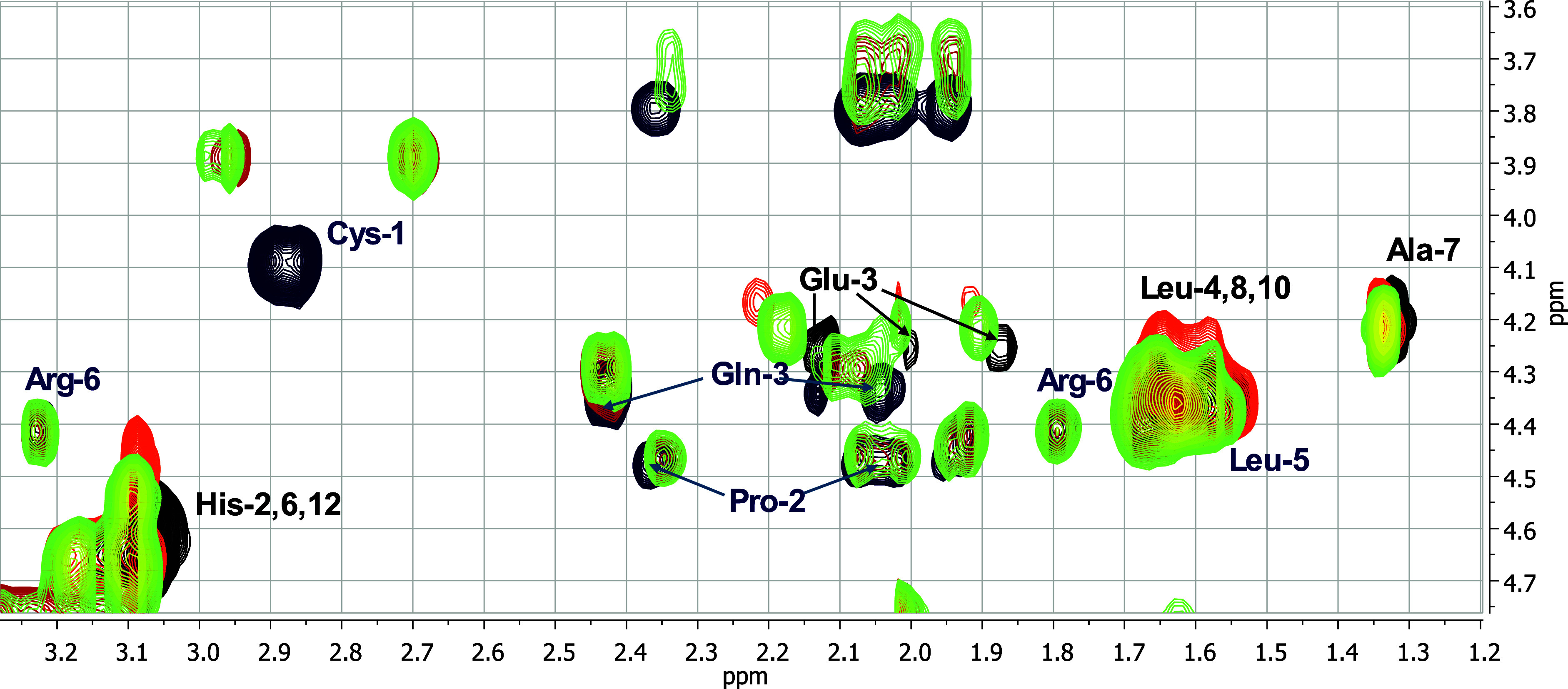
Superimposition of ^1^H–^1^H TOCSY spectra of apo-MMP-1 (black), apo-Inh4 (navy blue), Zn­(II)-MMP-1 (orange, *C*_*MMP‑1*_ = 0.8 mM, molar ratio Zn­(II):MMP-1 0.7:1), Zn­(II)-Inh4 (red, *C*_*Inh4*_ = 0.8 mM, molar ratio Zn­(II):Inh4 0.7:1), and the MMP-1-Zn­(II)-Inh4 ternary complex (green, *C*_*MMP‑1*_ = 0.8 mM, molar ratio Zn­(II):MMP-1:Inh4 = 0.7:1:1) at pH 7.55.

### MMP-1-Zn­(II)-Inh2’ System

Analysis of ZnAL ternary complex distribution diagrams (A = **MMP-1,** L = **Inh2’**) shows that at low pH, the binary Zn­(II)­A and the ternary species [ZnH_2_AL] are in equilibrium (Figure S12). [ZnH_2_AL] species appears at pH 5 and reaches its maximum at pH 6.4. As the pH increases this complex form is replaced by [ZnHAL], and subsequently by the [ZnAL] species. The formation of [ZnHAL] (p*K*_a_ = 6.86) likely corresponds to the deprotonation and coordination of the last histidine from the **MMP-1** ligand, while the transition to [ZnAL] (p*K*_a_ = 7.04) is attributed to cysteine coordination from **Inh2’** (Table 5). At more alkaline pH, deprotonation of coordinated water molecules leads to the formation of the [ZnH_–1_AL] and [ZnH_–2_AL] species (Figure S12). The formation of [ZnH_–1_L], and [ZnH_–2_L] species suggests that, at higher pH, **Inh2’** can also bind Zn­(II) independently. However, from pH 7 onward, coinciding with its optimal catalytic activity, **MMP-1** is predominantly present in ternary complexes similar to the **MMP-1**-Zn­(II)-**Inh4** system. NMR spectrum of the **MMP-1**-Zn­(II)-**Inh2’** system was recorded at pH 7.55 (Figure S14). Comparison with the corresponding binary complexes revealed, as in the case of **MMP-1**-Zn­(II)-**Inh4** complex, characteristic chemical shift variations of histidine and Glu-1 residues from **MMP-1**, confirming newly formed coordination environment (Figure S14). In addition, a new shift of the Cys-5 Hα-Hβ cross-peaks from **Inh2’** was detected, directly confirmed the involvement of this residue in Zn­(II) binding within the ternary complex. Interestingly, the perturbations observed for Pro-1, Gln-2, and Leu-4 upon Zn­(II) binding to the **Inh2′** peptide in a binary complex were absent in the ternary spectrum (Figure S10, Figure S14). This indicates that structural rearrangements of **Inh2′**, which occur in the binary complex to optimize Zn­(II) binding geometry, are suppressed in the ternary system, where Cys-5 directly coordinates through thiol to the {3N_im_} coordination mode of **MMP-1.**

**Table 5:** Stability Constants and Parameters of Ternary Complex of [ZnAL] Species of MMP-1 (A) with Analyzed Inhibitors (L)

inhibitor	**logβ**_*MAL*_^*M*^	**log** *K*_*MAL*_^*MA*^	**log** *K*_*MAL*_^*ML*^	Δlog*K*	%R.S
Inh4	13.91	8.80	6.36	1.25	16.56
Inh2’	12.07	6.96	4.94	–0.17	–2.38
Inh4’	12.33	7.22	5.08	–0.03	–0.42

### MMP-1-Zn­(II)-Inh4’ System

At acidic pH, binary complex species (A = **MMP-1,** L = **Inh4’**) form simultaneously, along with the first ternary species [ZnH_2_AL] (Figure S13). The next ternary species, [ZnHAL], begins to form at pH 6, reaches its maximum concentration around pH 7.2, and is rapidly substituted by the next form [ZnAL]. The p*K*_*a*_ of transition (p*K*_a_ = 6.76) corresponds to the deprotonation and coordination of a histidine residue from the **MMP-1** ligand, while the following transition to [ZnAL] (p*K*_a_ = 7.66) is attributed to cysteine from the inhibitor (Table 5). Above pH 8, [ZnAL] gradually transitions to [ZnH_–1_AL]. Beyond pH 8, **Inh4’** forms binary complex species – [ZnH_–1_L] and [ZnH_–2_L]. As observed in previous cases, from pH 6 onward, within the range of **MMP-1** highest catalytic activity (pH = 7.2–7.7), ternary complexes dominate in terms of formation and stability (Figure S13). NMR measurements for the **MMP-1**-Zn­(II)-**Inh4’** ternary system at pH 7.55 likewise confirmed the formation of a ternary complex (Figure S15). In this case, perturbations of histidine and Glu-1 residues from **MMP-1** were less pronounced compared to the previously studied systems. By contrast, a marked perturbation of the Cys-7 Hα-Hβ cross-peaks was observed, providing direct evidence for its involvement in Zn­(II) coordination and supporting a {3N_im_, S^–^} coordination mode in the ternary system.

### Comparison of Ternary Complexes

Given that both **MMP-1** and the inhibitors contain multiple protonation sites, the coordination process is pH-dependent and is more likely to proceed *via* the sequential mechanism, with formation of binary complexes followed by addition of the second ligand, rather than by a single simultaneous binding step. To determine which ligand acts as primary, and which one as a secondary ligand, the stepwise stability constants log*K*_*MAL*_^*MA*^ and log*K*_*MAL*_^*ML*^ were calculated for each mixed ligand system and compared (Table 5).^38–41^

Across all three systems, log*K*_*MAL*_^*MA*^ is consistently higher than log*K*_*MAL*_^*ML*^, indicating that the ternary complex formation is thermodynamically more favorable when **MMP-1** binds first Zn­(II) and the corresponding inhibitor coordinates in the second step. The **MMP-1** peptide functions as the primary ligand across all ternary systems, reflecting its expected role in the full-length enzyme. This supports the use of the catalytic domain as a reliable model for studying **MMP-1**-Zn­(II)-inhibitor interactions. One of the most important parameters used to indicate the stabilization of ternary complexes relative to their binary analogues is Δlog K value (Table 5).^38–40^ The statistical value of ΔlogK for a system with one tridentate A and one monodentate L ligands is −0.6 for tetrahedral complexes, reflecting the reduced number of coordination sites available for the second ligand. Therefore, when the experimentally observed Δlog K exceeds this statistical value, it indicates an additional stabilization effect on the ternary complexes.^66,67^ Another parameter, percent relative stabilization (%R.S) quantifies extent of this stabilization (or destabilization), based on the Δlog*K.* Among the investigated systems, **MMP-1**-Zn­(II)-**Inh4** exhibits the highest stability with ΔlogK = 1.25, and %R. S = 16.56%, exceeding the expected statistical limit. This suggests the additional stabilizing interactions in the ternary complex and the strongest preference for ternary complex formation over corresponding binary species. Both, **MMP-1**-Zn­(II)-**Inh2’** with a ΔlogK = −0.17 (%R.S = −2.38%) and **MMP-1**-Zn­(II)-**Inh4**’ with a ΔlogK = −0.03 (R.S = −0.42%), exhibit ΔlogK higher than −0.6, however lower than in case of **Inh4** (Table 5). These findings provide valuable insight into how atom donor’s positioning and steric factors govern metal–ligand interactions in both binary and ternary systems.

To elucidate the structural and energetic properties of the ternary complexes, we conducted a series of ONIOM (DFT/AM1) calculations. These revealed three Zn­(II)-**MMP-1** complexes with **Inh2’**, **Inh4’** and **Inh4** ligands distinct but structurally related coordination states (Figure 9).

**Figure 9:**
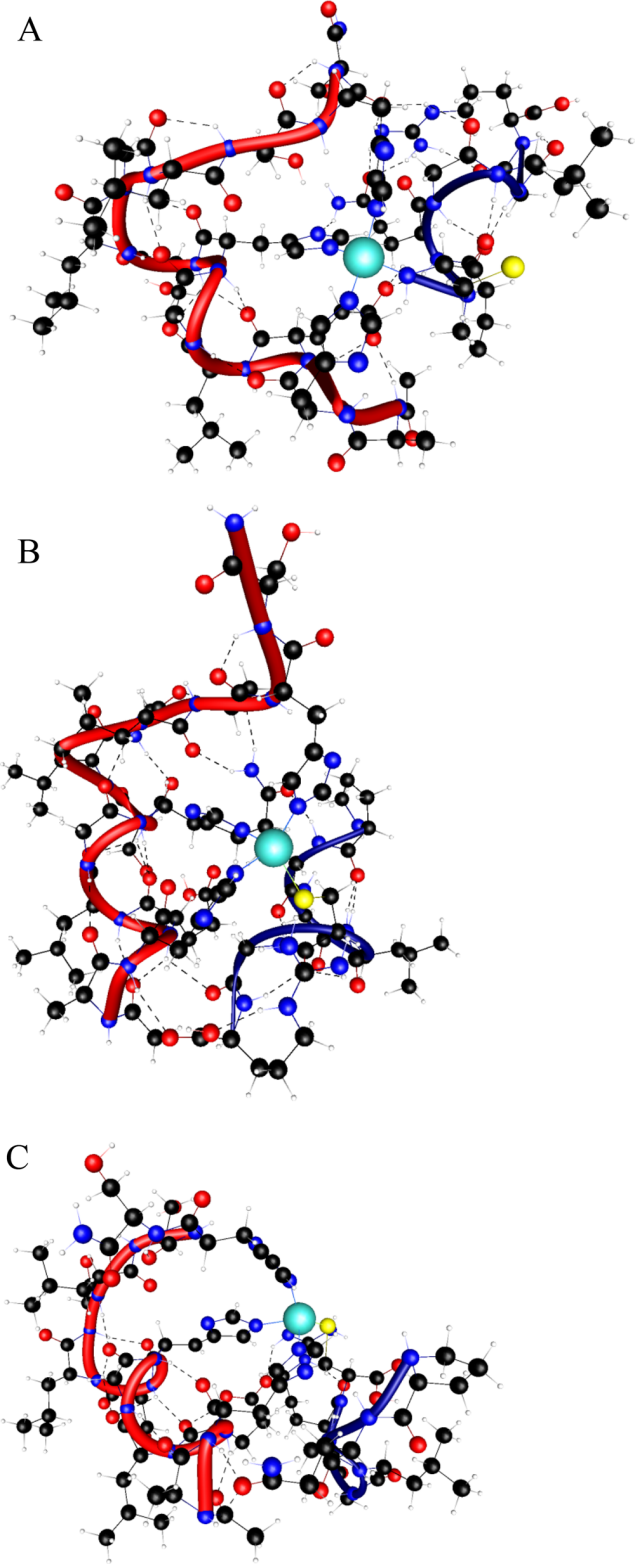
Structures of the complexes: (A) MMP-1···Zn­(II) ···Inh4, (B) MMP-1···Zn­(II) ···Inh2’, and (C) MMP-1···Zn­(II) ···Inh4’ complexes. MMP-1 ligand (red), respective inhibitor (blue), and Zn­(II) (cyan sphere).

Analysis of the coordination data reveals two distinct binding patterns. While all three **MMP-1**···Zn­(II) complexes maintain the characteristic imidazole pocket coordination, the **MMP-1**···Zn­(II)···**Inh4** complex is uniquely distinguished by interaction with the terminal amino group, exhibiting a metal–nitrogen distance of 2.084 Å. In contrast, the **Inh4’** and **Inh2’** ligands coordinate with the metal center via the thiolate sulfur of the cysteine residue. This differential binding pattern correlates with distinct complex stabilities, which decrease in the following order: **MMP-1**···Zn­(II)···**Inh4** ≫ **MMP-1**···Zn­(II)···**Inh4’**> **MMP-1**···Zn­(II)···**Inh2’**. Notably, the Zn···S bond distances of 2.269 Å (**MMP-1**···Zn­(II)···**Inh4’**) and 2.360 Å (**MMP-1**···Zn­(II)···**Inh2’**) show a clear correlation with complex stability - the longer bond distance in **MMP-1**···Zn­(II)···**Inh2’** (∼0.1 Å) corresponding to its relatively weaker stability compared to **MMP-1**···Zn­(II)···**Inh4’**, consistent with the overall stability trend. The distinctive **MMP-1**···Zn­(II)···NH_2_ binding pattern observed in the **MMP-1**···Zn­(II)···**Inh4** complex confers superior thermodynamic stability compared to all other complexes in the series, the coordination mode involving **MMP-1**···Zn­(II)···NH_2_ interaction in **MMP-1**···Zn­(II)**···Inh4** (metal–nitrogen distance = 2.084 Å) establishes a particularly stable binding pattern, explaining its enhanced thermodynamic stability relative to both the sulfur-coordinated **MMP-1**···Zn­(II)**···Inh4’** and **MMP-1**···Zn­(II)**···Inh2’** complexes. The complete set of coordination bond distances, including Zn­(II)-ligand interactions and key structural parameters for all complexes is summarized in Table 6. These metrics provide quantitative insight into the observed stability trends and binding mode differences discussed above.

**Table 6:** Bond Lengths (Å) between the Zinc Center and Coordinating Ligands in the MMP-1 Complexes

	ligand		
	Inh4	Inh4’	Inh2’
N_MMP‑1_(His2)···Zn(II)	1.950	1.976	1.938
N_MMP‑1_(His6)···Zn(II)	2.000	2.021	2.054
N_MMP‑1_(His12)···Zn(II)	1.959	2.014	2.028
N(N-term)···Zn(II)	2.084		
S(Cys)···Zn(II)		2.269	2.360

To summarize the theoretical calculations, it is important to note that all **MMP-1**···Zn­(II)···ligand complexes retain the conserved triple-imidazole···Zn­(II) coordination motif characteristic of metalloproteinase active sites. Two distinct binding modes were identified: Class I: Features symmetric S­(Cys)···Zn­(II)···S­(Cys) bridges (observed in Inh4’ and Inh2’ complexes; Zn–S bond distances ∼ 2.3–2.4 Å), class II: Incorporates an additional N-terminus-Zn­(II) interaction (unique to Inh4; Zn–N distance ∼ 2.1 Å). In addition to the **MMP-1**-bound complexes, the **Inh4’**, **Inh2’**, and **Inh4** ligands form bis­(ligand) structures interconnected by Zn­(II), with cysteine thiolates serving as bridging ligands. Notably, the N-terminal positioning of cysteine in **Inh4** enables supplementary amino–metal interactions, conferring enhanced stability compared to other complexes in the series. The stability hierarchy across the series follows a consistent trend where complexes containing **Inh4** are the most stable in both series. The **Inh2’** complexes show the lowest stability due to suboptimal cysteine positioning (central chain location).

## Conclusions

This study examined the interactions between peptide-based inhibitors (**Inh4, Inh2′, Inh4′**) and Zn­(II) ions, as well as between the Zn­(II)-**MMP-1** catalytic domain. **MMP-1**, which is strongly linked to cancer progression, requires Zn­(II) and a nucleophilic water molecule in its active site for enzymatic function. The inhibitors were designed as substrate-mimicking peptides with a cysteine residue introduced at different positions to act as a zinc-binding group (ZBG). The aim was to evaluate the influence of the cysteine residue position on the stability of the ternary complexes and their potential to suppress **MMP-1** activity.

All inhibitors formed both monomeric and bis­(ligand) Zn­(II) complexes, with **Inh4** showing the highest thermodynamic stability, whereas **Inh2′** and **Inh4′** required conformational rearrangements for an effective Zn­(II) coordination. Potentiometric analysis confirmed that the **MMP-1** active site maintained its characteristic {3N_im_} coordination in both binary and ternary systems, validating its use as a reliable model for early stage inhibitor studies.

In all ternary systems, **MMP-1** acts as the primary Zn­(II) ligand, with inhibitors binding in the second coordination step. At physiological pH, ternary complexes predominate, coinciding with the enzyme’s activity range and underscoring their biological relevance. Among the inhibitors, **Inh4** exhibited the greatest stabilization of ternary complexes (ΔlogK = 1.25; %R.S = 16.56%). This enhanced stability is attributed to the positioning of cysteine at the N-terminus, which provides accessible donor atoms (N-terminal amine and thiol). While NMR could not resolve whether the thiol or amine dominates coordination in the ternary system, DFT calculations supported binding through the N-terminal nitrogen. This mechanism parallels the inhibitory strategy of tissue inhibitors of metalloproteinases (TIMPs), where Cys-1 coordinates Zn­(II) *via* the amino and carbonyl groups, though in the present case carbonyl involvement was not detected.^68–70^ By contrast, **Inh2′** and **Inh4′**, which coordinate *via* cysteine thiols in ternary systems, formed less thermodynamic stable complexes, likely due to steric constraints.

Overall, these results identify N-terminal cysteine as the most effective ZBG placement, providing dual donor availability and enhancing ternary complex stability. **Inh4** therefore emerges as the most promising candidate for **MMP-1** inhibition, although further studies on the full-length enzyme and in biological assays will be essential to validate its therapeutic potential.

## Supplementary Material



## Data Availability

All data supporting the findings of this study, including raw potentiometric titrations, NMR spectra, mass spectrometry data, and DFT calculation files, are openly available in the RODBUK repository at https://doi.org/10.34616/NUNVEJ.

## Notes

Complementary potentiometric data, DFT calculations, additional ESI-MS, and NMR spectra of the studied systems, species distribution diagrams (PDF)

## References

[1] Ferlay, J. ; Ervik, M. ; Lam, F. ; Colombet, M. ; Mery, L. ; Piñeros, M. Global Cancer Observatory: Cancer Today; International Agency for Research on Cancer: Lyon, 2020.

[2] Bray F., Laversanne M., Sung H., Ferlay J., Siegel R. L., Soerjomataram I., Jemal A. (2024). Global Cancer Statistics 2022: GLOBOCAN
Estimates of Incidence and Mortality Worldwide for 36 Cancers in 185
Countries. CA: A Cancer Journal for Clinicians.

[3] Seyfried T.
N., Huysentruyt L. C. (2013). On the
Origin of Cancer Metastasis. Crit Rev. Oncog.

[4] Guan X. (2015). Cancer Metastases:
Challenges and Opportunities. Acta Pharmaceutica
Sinica B.

[5] Mansouri V., Beheshtizadeh N., Gharibshahian M., Sabouri L., Varzandeh M., Rezaei N. (2021). Recent Advances in
Regenerative Medicine Strategies
for Cancer Treatment. Biomedicine & Pharmacotherapy.

[6] Pucci C., Martinelli C., Ciofani G. (2019). Innovative Approaches for Cancer
Treatment: Current Perspectives and New Challenges. Ecancermedicalscience.

[7] Khan M. I., Hossain M. I., Hossain M. K., Rubel M. H. K., Hossain K. M., Mahfuz A. M. U. B., Anik M. I. (2022). Recent Progress in Nanostructured
Smart Drug Delivery Systems for Cancer Therapy: A Review. ACS Appl. Bio Mater..

[8] Gonzalez-Avila G., Sommer B., Mendoza-Posada D. A., Ramos C., Garcia-Hernandez A. A., Falfan-Valencia R. (2019). Matrix Metalloproteinases
Participation in the Metastatic
Process and Their Diagnostic and Therapeutic Applications in Cancer. Critical Reviews in Oncology/Hematology.

[9] Quintero-Fabián S., Arreola R., Becerril-Villanueva E., Torres-Romero J. C., Arana-Argáez V., Lara-Riegos J., Ramírez-Camacho M. A., Alvarez-Sánchez M. E. (2019). Role of Matrix Metalloproteinases
in Angiogenesis and Cancer. Front Oncol.

[10] Mondal S., Adhikari N., Banerjee S., Amin S. A., Jha T. (2020). Matrix Metalloproteinase-9
(MMP-9) and Its Inhibitors in Cancer: A Minireview. Eur. J. Med. Chem..

[11] Gobin E., Bagwell K., Wagner J., Mysona D., Sandirasegarane S., Smith N., Bai S., Sharma A., Schleifer R., She J.-X. (2019). A Pan-Cancer Perspective of Matrix
Metalloproteases
(MMP) Gene Expression Profile and Their Diagnostic/Prognostic Potential. BMC Cancer.

[12] Min M. W., Kim C.-E., Chauhan S., Park H. J., Park C.-S., Yoo T. H., Kang T. J. (2019). Identification of
Peptide Inhibitors
of Matrix Metalloproteinase 1 Using an In-House Assay System for the
Enzyme. Enzyme Microb Technol..

[13] Huang H. (2018). Matrix Metalloproteinase-9
(MMP-9) as a Cancer Biomarker and MMP-9 Biosensors: Recent Advances. Sensors.

[14] Shi Y., Ma X., Fang G., Tian X., Ge C. (2021). Matrix Metalloproteinase
Inhibitors (MMPIs) as Attractive Therapeutic Targets: Recent Progress
and Current Challenges. NanoImpact.

[15] Cabral-Pacheco G. A., Garza-Veloz I., Castruita-De la Rosa C., Ramirez-Acuña J. M., Perez-Romero B. A., Guerrero-Rodriguez J.
F., Martinez-Avila N., Martinez-Fierro M. L. (2020). The Roles of Matrix Metalloproteinases and Their Inhibitors
in Human Diseases. Int. J. Mol. Sci..

[16] Laronha H., Caldeira J. (2020). Structure and Function
of Human Matrix Metalloproteinases. Cells.

[17] Visse R., Nagase H. (2003). Matrix Metalloproteinases
and Tissue Inhibitors of
Metalloproteinases: Structure, Function, and Biochemistry. Circ. Res..

[18] Nagase H., Visse R., Murphy G. (2006). Structure
and Function of Matrix
Metalloproteinases and TIMPs. Cardiovasc. Res..

[19] Niland S., Riscanevo A. X., Eble J. A. (2022). Matrix Metalloproteinases Shape the
Tumor Microenvironment in Cancer Progression. Int. J. Mol. Sci..

[20] Nagase H. (2001). Metalloproteases. Curr. Protoc. Protein Sci..

[21] Gona K., Toczek J., Ye Y., Sanzida N., Golbazi A., Boodagh P., Salarian M., Jung J.-J., Rajendran S., Kukreja G., Wu T. L., Devel L., Sadeghi M. M. (2020). Hydroxamate-Based
Selective Macrophage Elastase (MMP-12) Inhibitors and Radiotracers
for Molecular Imaging. J. Med. Chem..

[22] Laronha H., Carpinteiro I., Portugal J., Azul A., Polido M., Petrova K. T., Salema-Oom M., Caldeira J. (2020). Challenges in Matrix
Metalloproteinases Inhibition. Biomolecules.

[23] Gattringer J., Gruber C. W., Hellinger R. (2023). Peptide Modulators
of Cell Migration:
Overview, Applications and Future Development. Drug Discovery Today.

[24] Maola K., Wilbs J., Touati J., Sabisz M., Kong X., Baumann A., Deyle K., Heinis C. (2019). Engineered Peptide
Macrocycles Can Inhibit Matrix Metalloproteinases with High Selectivity. Angew. Chem., Int. Ed..

[25] Wu D., Gao Y., Qi Y., Chen L., Ma Y., Li Y. (2014). Peptide-Based
Cancer Therapy: Opportunity and Challenge. Cancer
Lett..

[26] Wang L., Wang N., Zhang W., Cheng X., Yan Z., Shao G., Wang X., Wang R., Fu C. (2022). Therapeutic
Peptides: Current Applications and Future Directions. Sig Transduct Target Ther.

[27] Jensen S. A., Andersen P., Vrhovski B., Weiss A. S. (2003). Rational Design
of Tropoelastin Peptide-Based Inhibitors of Metalloproteinases. Arch. Biochem. Biophys..

[28] Hu J., Van den Steen P. E., Dillen C., Opdenakker G. (2005). Targeting
Neutrophil Collagenase/Matrix Metalloproteinase-8 and Gelatinase B/Matrix
Metalloproteinase-9 with a Peptidomimetic Inhibitor Protects against
Endotoxin Shock. Biochem. Pharmacol..

[29] Amar S., Smith L., Fields G. B. (2017). Matrix Metalloproteinase Collagenolysis
in Health and Disease. Biochim. Biophys. Acta,
Mol. Cell Res..

[30] Kridel S. J., Chen E., Kotra L. P., Howard E. W., Mobashery S., Smith J. W. (2001). Substrate Hydrolysis by Matrix Metalloproteinase-9*. J. Biol. Chem..

[31] Fields G. B. (2000). Using Fluorogenic
Peptide Substrates to Assay Matrix Metalloproteinases. Methods Mol. Biol..

[32] Neumann U., Kubota H., Frei K., Ganu V., Leppert D. (2004). Characterization
of Mca-Lys-Pro-Leu-Gly-Leu-Dpa-Ala-Arg-NH2, a Fluorogenic Substrate
with Increased Specificity Constants for Collagenases and Tumor Necrosis
Factor Converting Enzyme. Anal. Biochem..

[33] Rawlings N. D., Barrett A. J., Thomas P. D., Huang X., Bateman A., Finn D. (2018). The MEROPS database of proteolytic enzymes, their substrates and
inhibitors in 2017 and a comparison with peptidases in the PANTHER
database. Nucleic Acids Res..

[34] Murali R., Zhang H., Cai Z., Lam L., Greene M. (2021). Rational Design
of Constrained Peptides as Protein Interface Inhibitors. Antibodies.

[35] Gran G., Dahlenborg H., Laurell S., Rottenberg M. (1950). Determination
of the Equivalent Point in Potentiometric Titrations. Acta Chem. Scand..

[36] Gans P., O’Sullivan B. (2000). GLEE, a New
Computer Program for Glass Electrode Calibration. Talanta.

[37] Gans P., Sabatini A., Vacca A. (1996). Investigation of Equilibria
in Solution.
Determination of Equilibrium Constants with the HYPERQUAD Suite of
Programs. Talanta.

[38] Orabi A. S., Abdelhameed M., Abbas A. M., Mostafa G. M. (2022). Modern View for
Binary and Ternary Complexes of Metal Ions with Amoxicillin and Some
Amino Acids. Adv. Environ. Life Sci..

[39] de
Miranda J. L., Felcman J. (2003). Study on Guanidino–Carboxylate
Interactions in Copper­(II) Ternary Complexes of Guanidinoacetic Acid
with Glutamic and Aspartic Acids. Polyhedron.

[40] Türkel N. (2015). Equilibrium
Study of the Mixed Complexes of Copper­(II) with Adenine and Amino
Acids in Aqueous Solution. J. Solution Chem..

[41] Alturiqi A. S., Al-Farraj E. S., Anazy M. M., Ammar R. A. (2022). Potentiometric Determination
of Stability Constants of Binary and Ternary Complexes of L-Tryptophan
and Anti-Inflammatory Drugs with Zn­(II). Int.
J. Electrochem. Sci..

[42] Türkel N. (2015). Stability
Constants of Mixed Ligand Complexes of Nickel­(II) with Adenine and
Some Amino Acids. Bioinorg. Chem. Appl..

[43] Giraud E., Araujo M. L., Del Carpio E., Lubes V. (2023). Speciation Study of
Nickel­(II)-1,10-Phenanthroline-Amino Acid Ternary Complexes in 1.0
M NaCl at 25 °C. J. Coord. Chem..

[44] Baes, C. F. ; Mesmer, R. E. The Hydrolysis of Cations; Wiley: 1976.

[45] Alderighi L., Gans P., Ienco A., Peters D., Sabatini A., Vacca A. (1999). Hyperquad Simulation
and Speciation (HySS): A Utility Program for
the Investigation of Equilibria Involving Soluble and Partially Soluble
Species. Coord. Chem. Rev..

[46] Gumienna-Kontecka E., Berthon G., Fritsky I. O., Wieczorek R., Latajka Z., Kozlowski H. (2000). 2-(Hydroxyimino)­Propanohydroxamic
Acid, a New Effective Ligand for Aluminium. J. Chem. Soc., Dalton Trans..

[47] Wieczorek R., Latajka Z., Lundell J. (1999). Quantum Chemical Study of the Bimolecular
Complex of HONO. J. Phys. Chem. A.

[48] Lesiów M. K., Komarnicka U. K., Stokowa-Sołtys K., Rolka K., Łęgowska A., Ptaszyńska N., Wieczorek R., Kyzioł A., Jeżowska-Bojczuk M. (2018). Relationship
between Copper (ii) Complexes with FomA Adhesin Fragments of *F. Nucleatum* and Colorectal Cancer. Coordination Pattern
and Ability to Promote ROS Production. Dalton
Trans..

[49] Miller A., Matera-Witkiewicz A., Mikołajczyk A., Wieczorek R., Rowińska-Żyrek M. (2021). Chemical “Butterfly Effect”
Explaining the Coordination Chemistry and Antimicrobial Properties
of Clavanin Complexes. Inorg. Chem..

[50] Wierzejewska M., Mielke Z., Wieczorek R., Latajka Z. (1998). Infrared Matrix Isolation
and Theoretical Studies of SO 2-HNO 3 and SO 2-HONO Systems. Chem. Phys..

[51] Wątły J., Hecel A., Wieczorek R., Świątek-Kozłowska J., Kozłowski H., Rowińska-Żyrek M. (2019). Uncapping
the N-Terminus of a Ubiquitous His-Tag Peptide Enhances Its Cu2+ Binding
Affinity. Dalton Trans..

[52] Latajka Z., Mielke Z., Olbert-Majkut A., Wieczorek R., Tokhadze K. G. (1999). Ab Initio Calculations and Matrix
Infrared Spectra
of the Nitrous Acid Complexes with HF and HCl. Phys. Chem. Chem. Phys..

[53] Dapprich S., Komáromi I., Byun K. S., Morokuma K., Frisch M. J. (1999). A New ONIOM
Implementation in Gaussian98. Part I. The Calculation of Energies,
Gradients, Vibrational Frequencies and Electric Field Derivatives1. Journal of Molecular Structure: THEOCHEM.

[54] Chai J.-D., Head-Gordon M. (2008). Long-Range
Corrected Hybrid Density Functionals with
Damped Atom–Atom Dispersion Corrections. Phys. Chem. Chem. Phys..

[55] Dewar M. J. S., Zoebisch E. G., Healy E. F., Stewart J. J. P. (1985). Development and
Use of Quantum Mechanical Molecular Models. 76. AM1: A New General
Purpose Quantum Mechanical Molecular Model. J. Am. Chem. Soc..

[56] Frisch, M. J. ; Trucks, G. W. ; Schlegel, H. B. ; Scuseria, G. E. ; Robb, M. A. ; Cheeseman, J. R. ; Scalmani, G. ; Barone, V. ; Petersson, G. A. ; Nakatsuji, H. ; Li, X. ; Caricato, M. ; Marenich, A. V. ; Bloino, J. ; Janesko, B. G. ; Gomperts, R. ; Mennucci, B. ; Hratchian, H. P. ; Ortiz, J. V. ; Izmaylov, A. F. ; Sonnenberg, J. L. ; Williams-Young, D. ; Ding, F. ; Lipparini, F. ; Egidi, F. ; Goings, J. ; Peng, B. ; Petrone, A. ; Henderson, T. ; Ranasinghe, D. ; Zakrzewski, V. G. ; Gao, J. ; Rega, N. ; Zheng, G. ; Liang, W. ; Hada, M. ; Ehara, M. ; Toyota, K. ; Fukuda, R. ; Hasegawa, J. ; Ishida, M. ; Nakajima, T. ; Honda, Y. ; Kitao, O. ; Nakai, H. ; Vreven, T. ; Throssell, K. ; Montgomery, Jr., J. A. ; Peralta, J. E. ; Ogliaro, F. ; Bearpark, M. J. ; Heyd, J. J. ; Brothers, E. N. ; Kudin, K. N. ; Staroverov, V. N. ; Keith, T. A. ; Kobayashi, R. ; Normand, J. ; Raghavachari, K. ; Rendell, A. P. ; Burant, J. C. ; Iyengar, S. S. ; Tomasi, J. ; Cossi, M. ; Millam, J. M. ; Klene, M. ; Adamo, C. ; Cammi, R. ; Ochterski, J. W. ; Martin, R. L. ; Morokuma, K. ; Farkas, O. ; Foresman, J. B. ; Fox, D. J. Gaussian 16, Revision B.01, Gaussian, Inc., Wallingford CT, 2016 GaussView 5.0. Wallingford, E.U.A. - References - Scientific Research Publishing. https://www.scirp.org/reference/referencespapers?referenceid=2418053 (accessed 2025–03–13).

[57] Árus D., Nagy N. V., Dancs Á., Jancsó A., Berkecz R., Gajda T. (2013). A Minimalist Chemical Model of Matrix
Metalloproteinases – Can Small Peptides Mimic the More Rigid
Metal Binding Sites of Proteins?. Journal of
Inorganic Biochemistry.

[58] Potok P., Kola A., Valensin D., Capdevila M., Potocki S. (2023). Copper Forms a PPII Helix-Like Structure
with the Catalytic
Domains of Bacterial Zinc Metalloproteases.. Inorg. Chem..

[59] Sóvágó I., Kiss T., Várnagy K., Révérend B. D.-L. (1988). Cobalt­(II)
and Zinc­(II) Complexes of Cysteine Containing Dipeptides.. Polyhedron.

[60] Gioia M., Fasciglione G. F., Monaco S., Iundusi R., Sbardella D., Marini S., Tarantino U., Coletta M. (2010). pH Dependence of the
Enzymatic Processing of Collagen I by MMP-1 (Fibroblast Collagenase),
MMP-2 (Gelatinase A), and MMP-14 Ectodomain. J. Biol. Inorg. Chem..

[61] Lu W., Zhu J., Zou S., Li X., Huang J. (2013). The Efficient Expression
of Human Fibroblast Collagenase in Escherichia Coli and the Discovery
of Flavonoid Inhibitors.. Journal of Enzyme
Inhibition and Medicinal Chemistry.

[62] Razaq S., Wilkins R. J., Urban J. P. G. (2003). The
Effect of Extracellular pH on
Matrix Turnover by Cells of the Bovine Nucleus Pulposus. Eur. Spine J..

[63] Lukács M., Csilla Pálinkás D., Szunyog G., Várnagy K. (2021). Metal Binding
Ability of Small Peptides Containing Cysteine Residues. ChemistryOpen.

[64] Raics M., Lihi N., Laskai A., Kállay C., Várnagy K., Sóvágó I. (2016). Nickel­(II),
Zinc­(II) and Cadmium­(II) Complexes of Hexapeptides Containing Separate
Histidyl and Cysteinyl Binding Sites.. New J.
Chem..

[65] Grenács Á., Lihi N., Sóvágó I., Várnagy K. (2017). The Influence of Penicillamine/Cysteine Mutation on
the Metal Complexes of Peptides. Dalton Trans..

[66] Sigel H. (1975). Ternary Cu2+
Complexes: Stability, Structure, and Reactivity. Angewandte Chemie International Edition in English.

[67] Sharma V. S., Schubert J. (1969). Statistical Factor
in the Formation and Stability of
Ternary and Mixed Complexes. J. Chem. Educ..

[68] Brew K., Nagase H. (2010). The Tissue Inhibitors
of Metalloproteinases (TIMPs):
An Ancient Family with Structural and Functional Diversity. Biochim. Biophys. Acta.

[69] Murphy G., Nagase H. (2008). Progress in Matrix
Metalloproteinase Research. Molecular Aspects
of Medicine.

[70] Liu J., Khalil R. A. (2017). Matrix Metalloproteinase Inhibitors as Investigational
and Therapeutic Tools in Unrestrained Tissue Remodeling and Pathological
Disorders. Prog. Mol. Biol. Transl Sci..

