# Supplementary material for: Substrate-Mimicking Peptides as MMP‑1 Inhibitors: Impact of Zinc-Binding Group Position on Ternary Complex Stability
Source: Inorg Chem. 2026 Jan 15;65(4):2203–16. doi: 10.1021/acs.inorgchem.5c04597 (PMC12869495; doi:10.1021/acs.inorgchem.5c04597)
Supplement: Supplementary file 1 [file ic5c04597_si_001.pdf]

Supporting Information

Substrate-Mimicking Peptides as MMP-1 Inhibitors: Impact of Zinc-Binding Group Position on Ternary Complex Stability

Paulina Potok[a], Wiktoria Woźniak-Laszczyńska[a], Robert Wieczorek[a], Merce Capdevila[b], Òscar Palacios[b], Elżbieta Gumienna-Kontecka[a], Sławomir Potocki*[a]

* Corresponding author, e-mail: slawomir.potocki@uwr.edu.pl
[a] Faculty of Chemistry, University of Wroclaw, 14 Joliot-Curie St., 50-383 Wroclaw, Poland
[b] Departament de Química, Universitat Autònoma de Barcelona, 08193 Cerdanyola del Vallès (Barcelona), Spain

Binary Zn(II) Complexes

Table S1 summarizes the stability constants (log β) and log(K_1/K_2) values for the Zn(II) complexes formed with the inhibitors. The log(K_1/K_2) values were calculated to evaluate the tendency of the inhibitors towards monomeric and bis(ligand) complex formation, discussed in the main manuscript.

Table S1. Stability constants (log*β*) and log(K_1/K_2) values for Zn(II) complexes formed with inhibitors **Inh4, Inh2′**, and **Inh4′**.

	**Inh4**	**Inh2'**	**Inh4'**
log*β* [ZnL]	7.55	7.15	7.25
log*β* [ZnL_2]	15.58	12.19	13.55
log(K_1/K_2)	-0.48	2.11	0.95

DFT calculations were performed to further examine the bis(ligand) complexes. **Table S2** presents the metal-ligand coordination distances, including Zn(II)-S(thiolate) and Zn(II)-N(amine) bond lengths, for all the characterized systems.

Table S2. Bond lengths (Å) between the zinc center and coordinating ligands in the Zn(II)···$(Ligand)_2$, complexes.

	**Ligand**		
	**Inh4**	**Inh4'**	**Inh2'**
**S(Cys_A)···Zn(II)**	2.255	2.264	2.336
**S(Cys_B)···Zn(II)**	2.259	2.155	2.216
**N(N-term$_A$)···Zn(II)**	2.118		
**N(N-term$_B$)···Zn(II)**	2.163		

The ESI-MS spectra for binary complexes of the tested inhibitors (Inh4, Inh2', Inh4') with Zn(II) are presented in the **Figures S1-S4**. In the attached ESI-MS spectra, we can observe a signal at 830.32 Da, confirming the formation of monomeric [ZnL]+ complexes (**Fig. S1-S3**). The experimental spectra matches the simulated ones, which confirms proper interpretation. Furthermore, when the inhibitor is present in excess (1:2 M:L ratio), additional peaks corresponding to bis(ligand) Zn(II) complexes appear (**Fig. S4**).

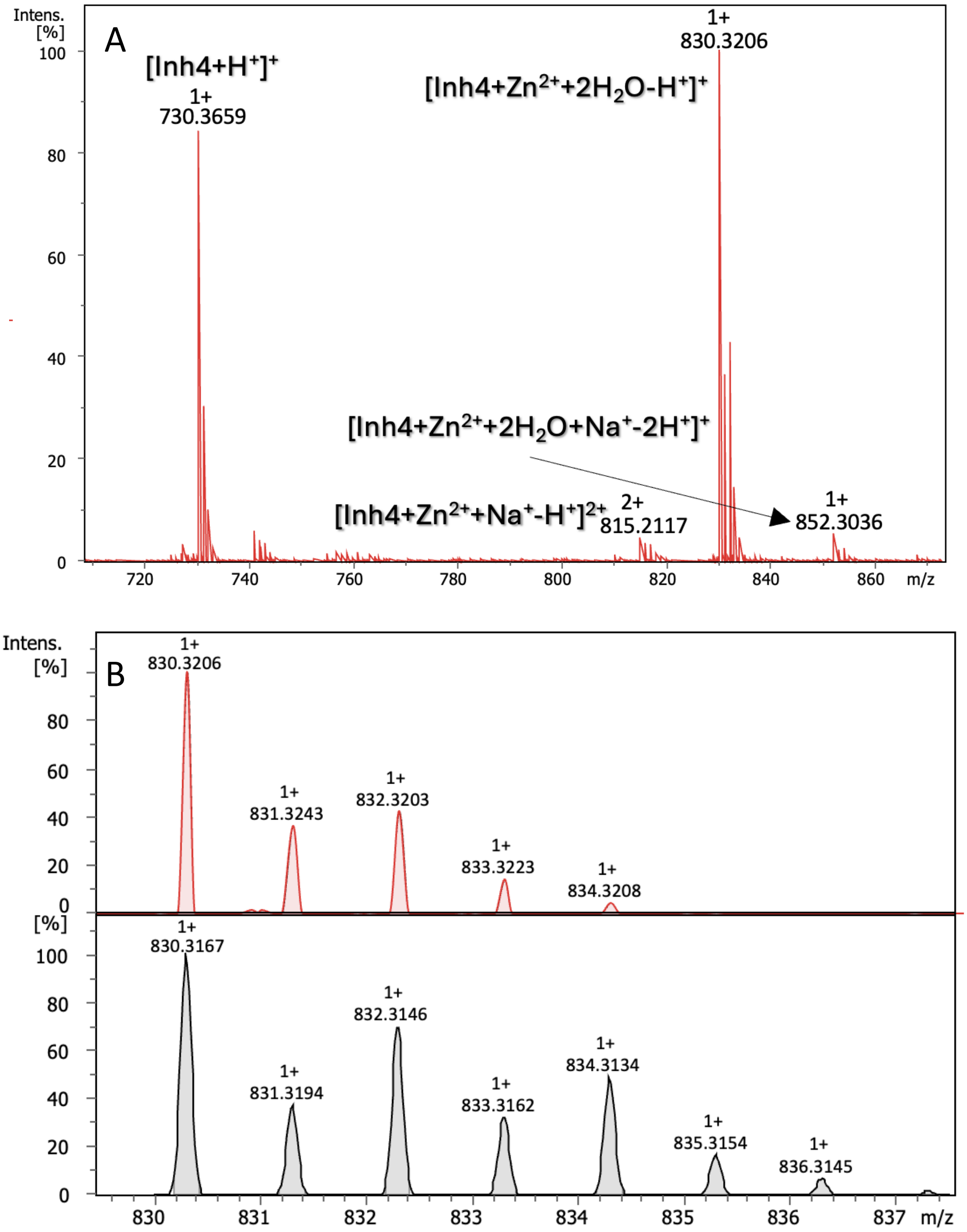

Figure S1. (A) ESI-MS spectrum of a system composed of the **Inh4** ligand and Zn(II) ions in the range of *m/z* 710-870 (1:1 M:L); (B) Experimental (red) and simulated (black) isotopic distribution of the signal at *m/z* 830.32 (z = 1), confirming the assignment of the $[ZnL]^+$ complex.

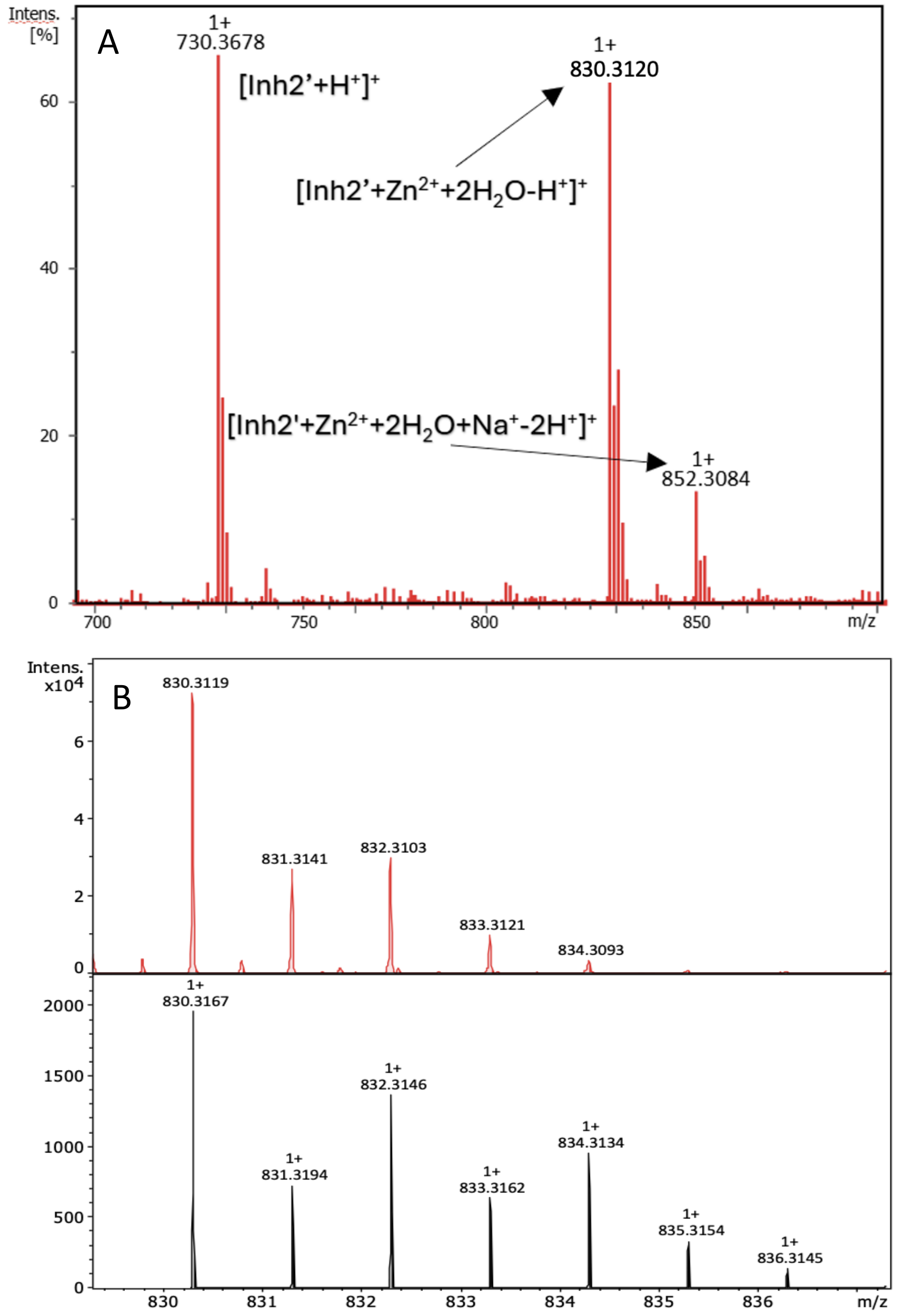

Figure S2. (A) ESI-MS spectrum of a system composed of the **Inh2'** and Zn(II) ions in the range of *m/z* 695-880 (1:1 M:L). (B) Experimental (red) and simulated (black) isotopic distribution of the signal at *m/z* 830.31 (z = 1), confirming the assignment of the $[ZnL]^+$ complex.

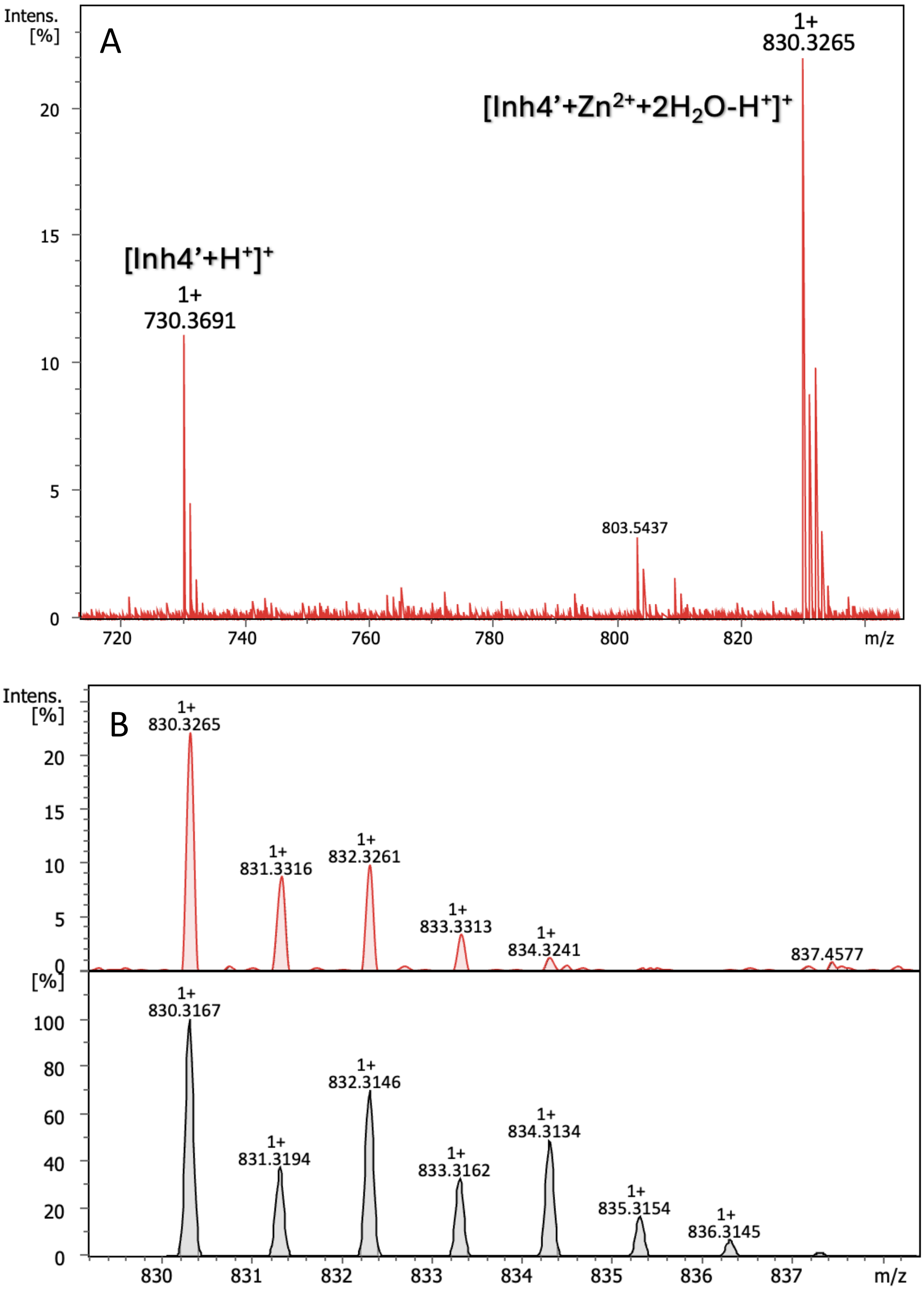

Figure S3. (A) ESI-MS spectrum of a system composed of the **Inh4'** and Zn(II) ions in the range of *m/z* 715-840 (1:1 M:L); (B) Experimental (red) and simulated (black) isotopic distribution of the signal at *m/z* 830.32 ($z = 1$), confirming the assignment of the $[ZnL]^+$ complex.

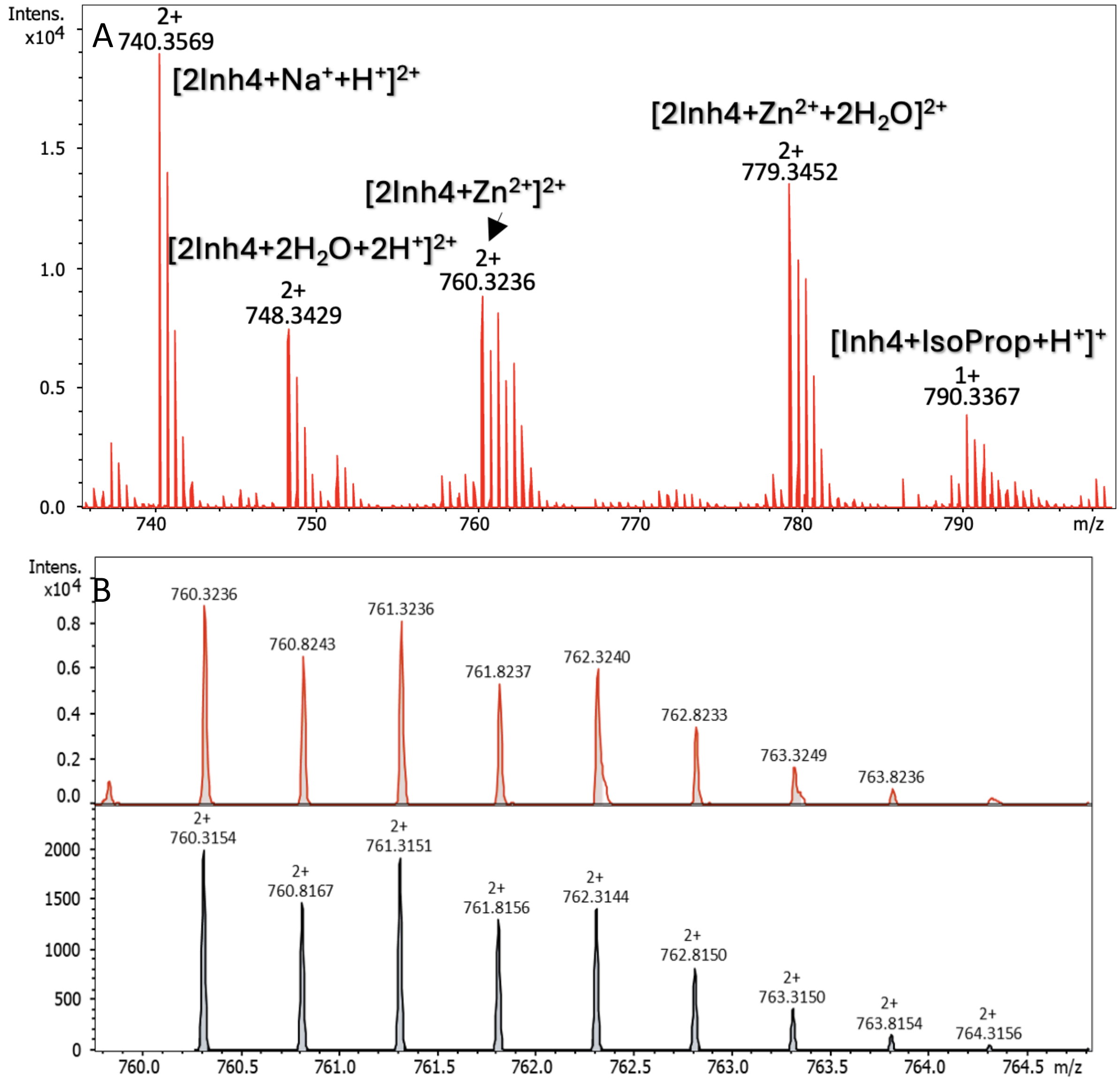

Figure S4. (A) ESI-MS spectrum of a system composed of the **Inh4** and Zn(II) ions in the range of *m/z* 735-800 (1:2 M:L); (B) Experimental (red) and simulated (black) isotopic distribution of the signal at *m/z* 760.32 ($z = 2$), confirming the assignment of the $[Zn(L)_2]^{2+}$ complex.

Figures S5, and **S7-S9** present species distribution diagrams for Zn(II) complexes with MMP-1 and the inhibitors, showing the relative abundance of Zn(II)-complex species as a function of pH. **Figure S6**, and **S10-S11**, show NMR spectra for MMP-1 and each inhibitor with and without Zn(II) ion. For the inhibitors, the NMR spectra were recorded for metal-free ligand and upon successive additions of Zn(II) (0.25, 0.5, 0.7, and 1.0 equivalents). The most significant spectral disturbances were observed in the cysteine residue peaks, which indicates its participation in metal coordination.

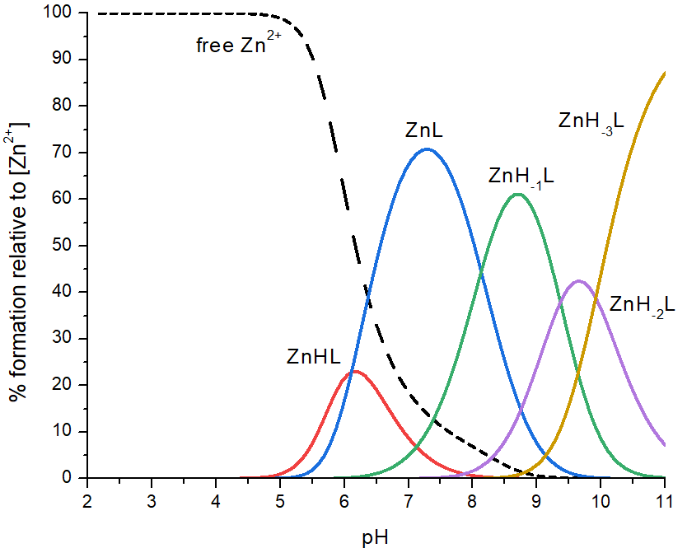

Figure S5. Species distribution diagram for Zn(II)-**MMP-1** complexes in an aqueous solution; C_L = 0.5 mM; molar ratio Zn(II):L = 0.5:1.

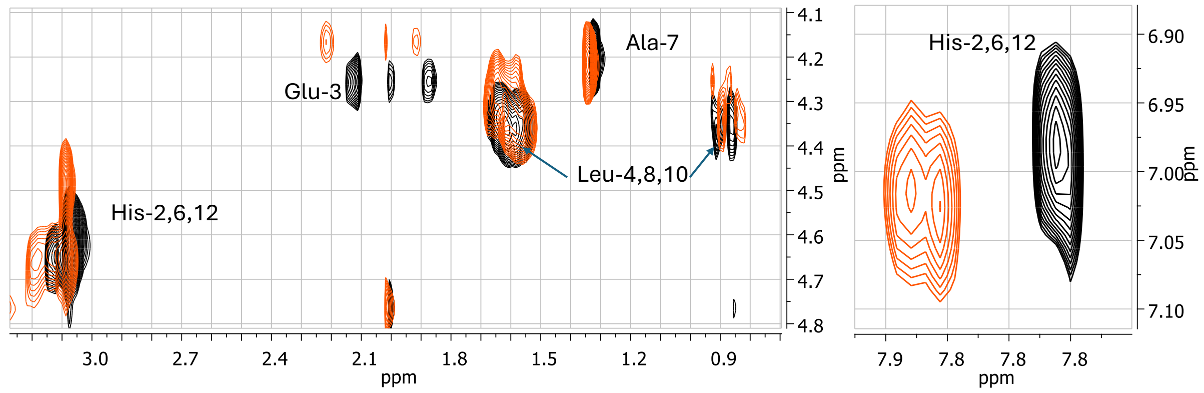

Figure S6. Superimposition of (A) aliphatic and (B) aromatic regions of ^1H–^1H TOCSY spectra of 0.8 mM **MMP-1**, pH 7.55, in the absence (black) and in the presence of 0.7 (orange) eq. of Zn(II).

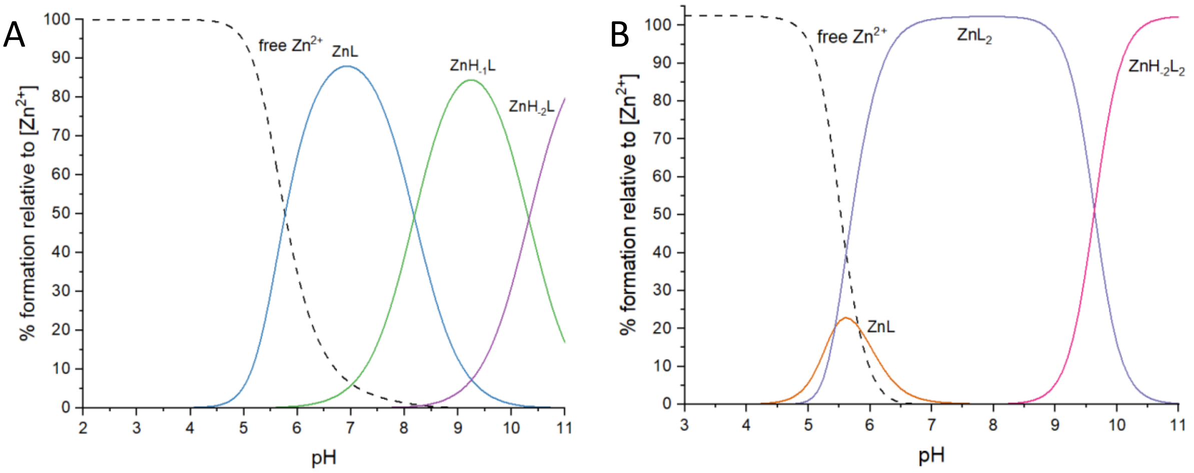

Figure S7. Species distribution diagram for Zn(II)-**Inh4** complexes in an aqueous solution; C_L = 0.5 mM; molar ratio Zn(II):L= (A) 1:1; (B) 1:2.

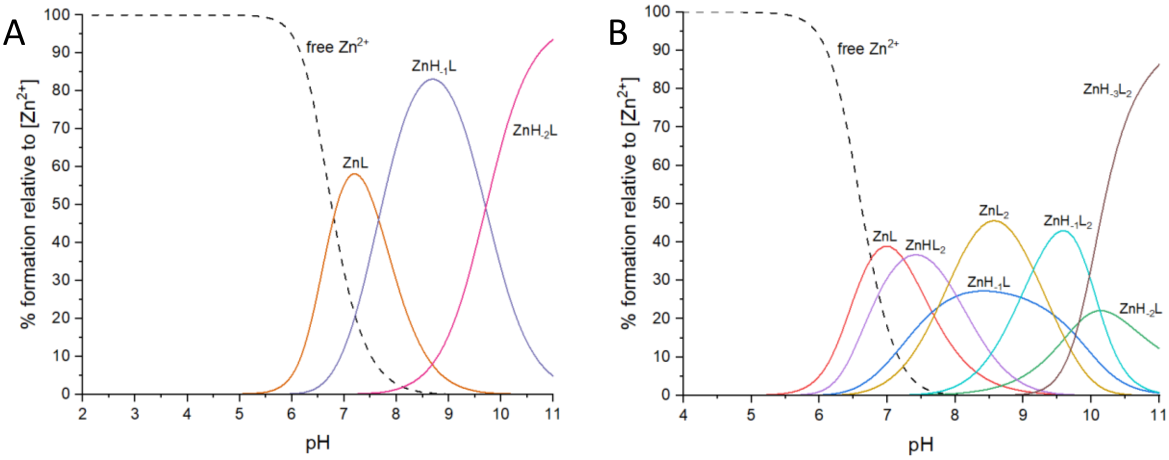

Figure S8. Species distribution diagram for Zn(II)-**Inh2'** complexes in an aqueous solution; C_L = 0.5 mM; molar ratio Zn(II):L= (A) 1:1; (B) 1:2

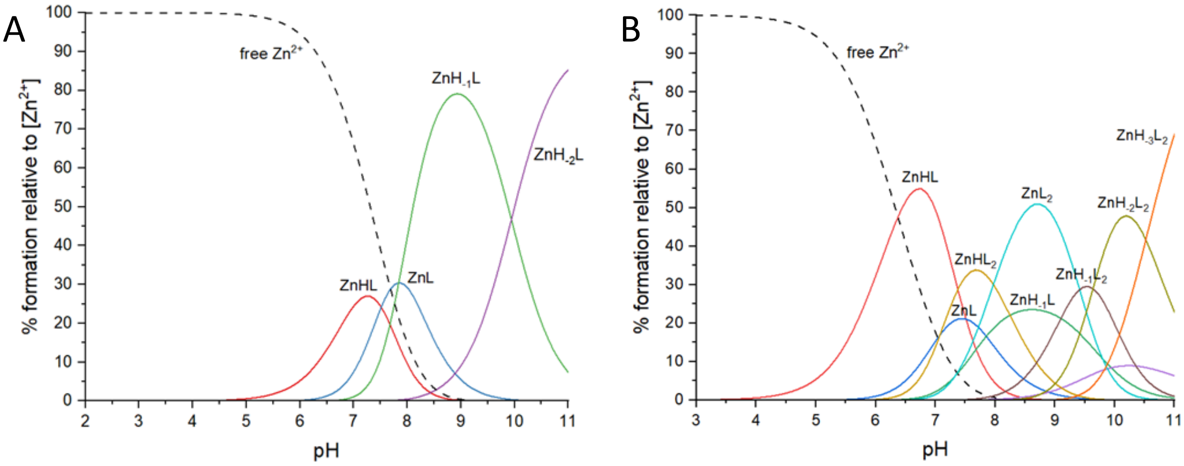

Figure S9. Species distribution diagram for Zn(II)-**Inh4'** complexes in an aqueous solution; C_L = 0.5 mM; molar ratio Zn(II):L= (A) 1:1; (B) 1:2

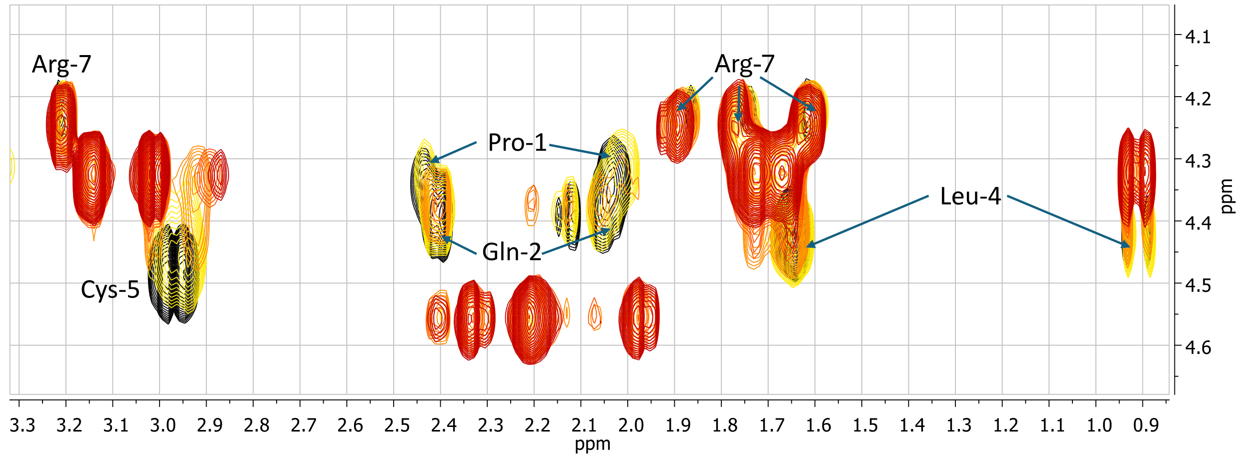

Figure S10. Superimposition of aliphatic regions of ^{1}H-^{1}H TOCSY spectra of 0.8 mM **Inh2'** peptide (PQGLCGR), pH 7.55, in the absence (black) and in the presence of increasing equivalents of Zn(II): 0.25 (yellow), 0.5 (orange), 0.7 (dark orange) and 1.0 (red).

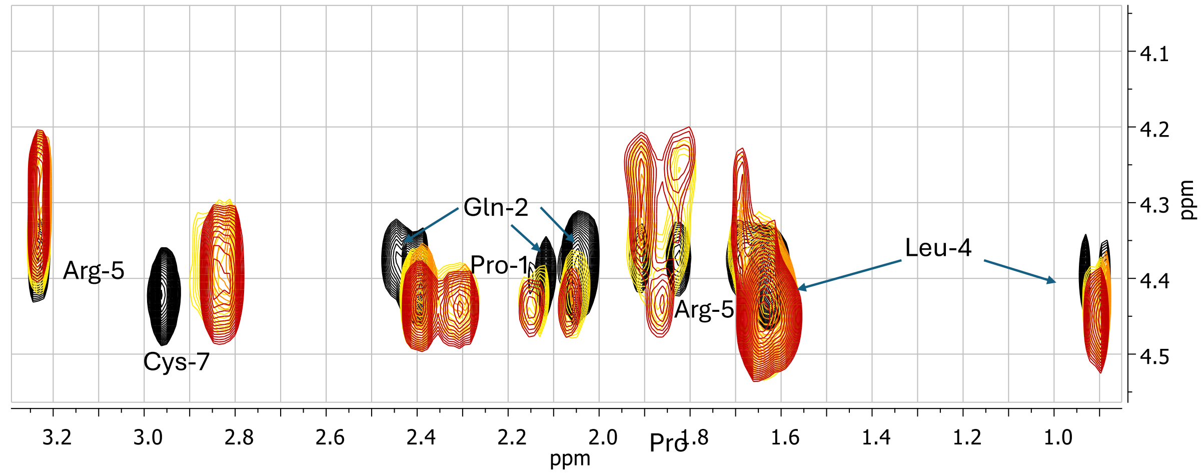

Figure S11. Superimposition of aliphatic regions of ^{1}H-^{1}H TOCSY spectra of 0.8 mM **Inh4'** peptide (PQGLRGC), pH 7.55, in the absence (black) and in the presence of increasing equivalents of Zn(II): 0.25 (yellow), 0.5 (orange), 0.7 (dark orange) and 1.0 (red).

Ternary Zn(II) Complexes

Potentiometric measurements enabled the determination of ternary complex species, their stability constants (logβ), and deprotonation constants (pKa) (**Tab. 4**), as well as the construction of species distribution diagrams presented below (**Figs. S12, S13**). Based on the species distribution diagrams, we can conclude that ternary complexes dominate over binary complexes across the entire pH range.

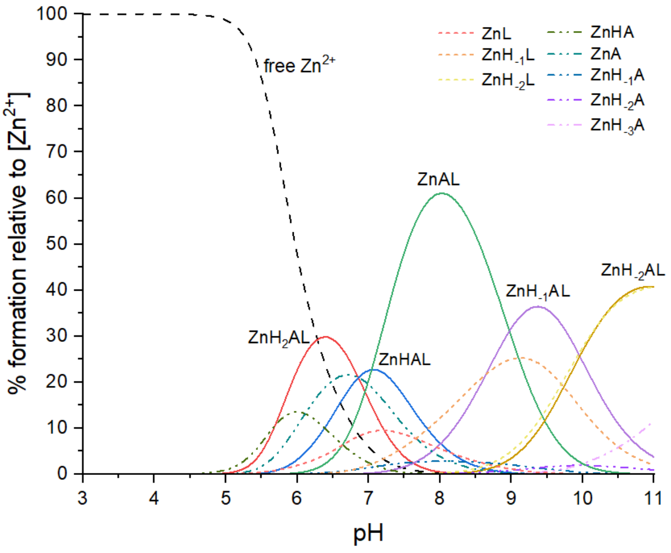

Figure S12. Species distribution diagram for **MMP-1**-Zn(II)-**Inh2'** complexes (A - **MMP-1**, L - **Inh2'**) in an aqueous solution; C_L = 0.5 mM; Zn(II):A:L = 1:1:1.

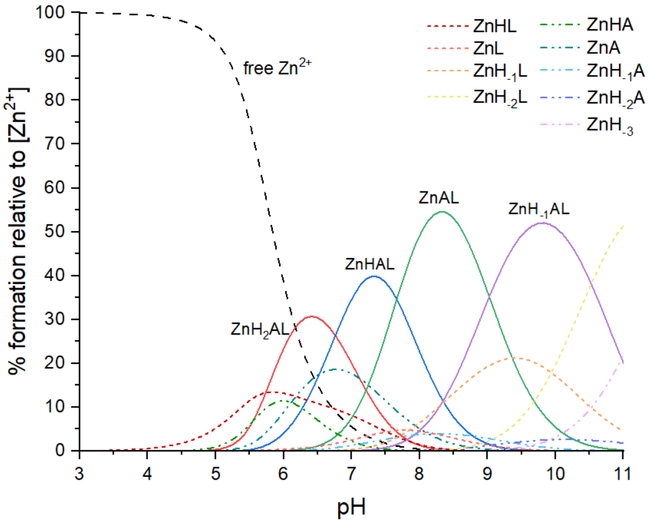

Figure S13. Species distribution diagram for **MMP-1**-Zn(II)-**Inh4'** complexes (A - **MMP-1**, L - **Inh4'**) in an aqueous solution; C_L = 0.5 mM; Zn(II):A:L = 1:1:1.

Figures **S14-S15** compare the ^{1}H-^{1}H TOCSY NMR spectra of binary and ternary systems involving MMP-1, Zn(II) ion and the inhibitor at pH 7.55, where ZnAL species predominate. The spectral changes observed for specific amino acids involved in the coordination provide evidence of ternary complex formation. In particular, histidine residues from MMP-1 show variation in their Hα-Hβ correlations compared to the binary Zn(II)-MMP-1 complex (**Fig. 8**), indicating their direct involvement in Zn(II)

coordination within a modified binding environment. Perturbations observed for the Cys-1 Hα-Hβ cross-peaks differ among the ternary complexes depending on the inhibitor and are discussed in detail in the main manuscript.

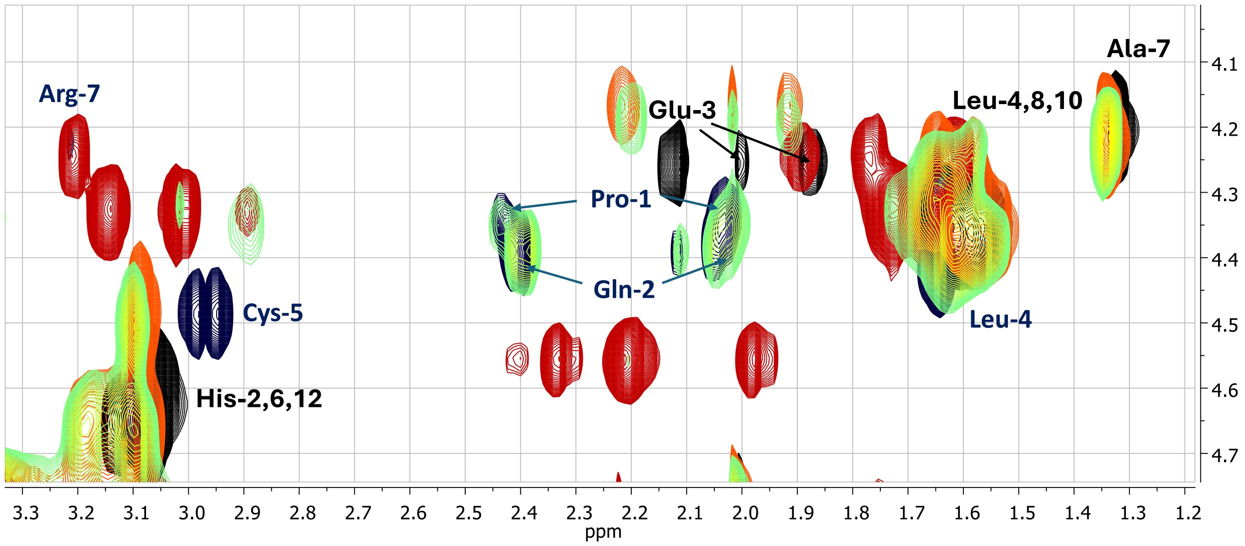

Figure S14. Superimposition of ^{1}H-^{1}H TOCSY spectra of apo-**MMP-1** (black), apo-**Inh2'** (navy blue), Zn(II)-**MMP-1** (orange, $C_{MMP\text{-}1}$ = 0.8 mM, molar ratio Zn(II):**MMP-1** 0.7:1), Zn(II)-**Inh2'** (red, $C_{Inh2'}$ = 0.8 mM, molar ratio Zn(II):**Inh4** 0.7:1), and the **MMP-1**-Zn(II)-**Inh2'** ternary complex (green, $C_{MMP\text{-}1}$ = 0.8 mM, molar ratio Zn(II):**MMP-1**:**Inh2'** = 0.7:1:1) at pH 7.55.

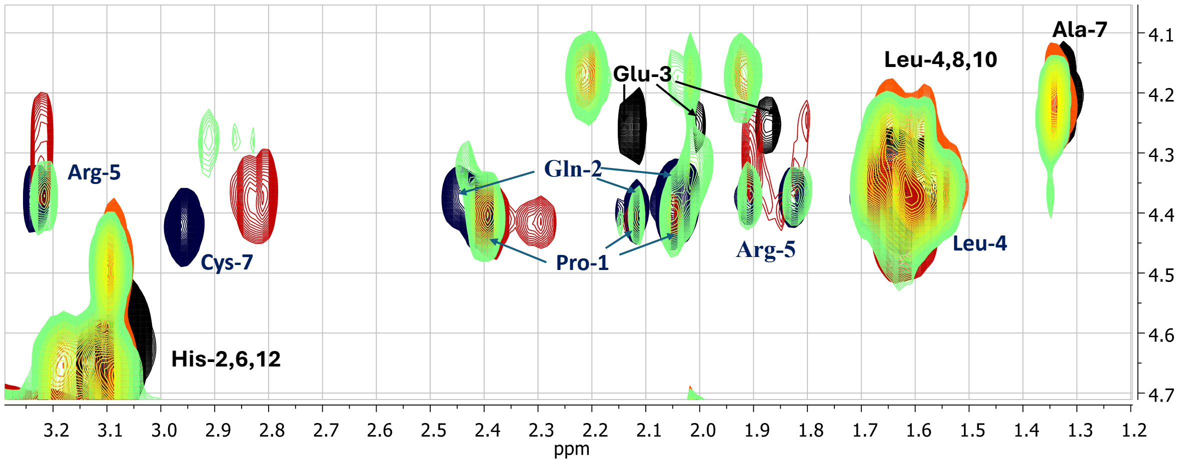

Figure S15. Superimposition of ^{1}H-^{1}H TOCSY spectra of apo-**MMP-1** (black), apo-**Inh4'** (navy blue), Zn(II)-**MMP-1** (orange, $C_{MMP\text{-}1}$ = 0.8 mM, molar ratio Zn(II):**MMP-1** 0.7:1), Zn(II)-**Inh4'** (red, $C_{Inh4'}$ = 0.8 mM, molar ratio Zn(II):**Inh4** 0.7:1), and the **MMP-1**-Zn(II)-**Inh4'** ternary complex (green, $C_{MMP\text{-}1}$ = 0.8 mM, molar ratio Zn(II):**MMP-1**:**Inh4'** = 0.7:1:1) at pH 7.55.
